# Consistent decreased activity in the putamen in Parkinson's disease: a meta-analysis and an independent validation of resting-state fMRI

**DOI:** 10.1093/gigascience/giy071

**Published:** 2018-06-18

**Authors:** Jue Wang, Jia-Rong Zhang, Yu-Feng Zang, Tao Wu

**Affiliations:** 1 Department of Neurobiology, Neurology and Geriatrics, Xuanwu Hospital of Capital Medical University, Institute of Geriatrics, No. 45, Changchun Rd, Xicheng District, 100053, Beijing, P. R. China; 2 Institutes of Psychological Sciences, Hangzhou Normal University, No. 2318, Yuhangtang Rd, Yuhang District, 311121, Hangzhou, P. R. China; 3 Zhejiang Key Laboratory for Research in Assessment of Cognitive Impairments, No. 2318, Yuhangtang Rd, Yuhang District, 311121, Hangzhou, P. R. China; 4 Center for Cognition and Brain Disorders and the Affiliated Hospital, Hangzhou Normal University, No. 2318, Yuhangtang Rd, Yuhang District, 311121, Hangzhou, P. R. China; 5 Clinical Center for Parkinson's Disease, Capital Medical University, No. 10, Youanmenwaixi Rd, Fengtai District, 100069, Beijing, P. R. China; 6 Key Laboratory for Neurodegenerative Disease of the Ministry of Education, Beijing Key Laboratory for Parkinson's Disease, Parkinson's Disease Center of Beijing Institute for Brain Disorders, No. 45, Changchun Rd, Xicheng District, 100053, Beijing, P. R. China; 7 National Clinical Research Center for Geriatric Disorders, No. 45, Changchun Rd, Xicheng District, 100053, Beijing, P. R. China; 8 Parkinson Disease Imaging Consortium of China (PDICC), No. 45, Changchun Rd, Xicheng District, 100053, Beijing, P. R. China

**Keywords:** Parkinson's disease, spontaneous brain activity, coordinate-based meta-analysis, putamen

## Abstract

**Background:**

Resting-state functional magnetic resonance imaging (RS-fMRI) has frequently been used to investigate local spontaneous brain activity in Parkinson's disease (PD) in a whole-brain, voxel-wise manner. To quantitatively integrate these studies, we conducted a coordinate-based (CB) meta-analysis using the signed differential mapping method on 15 studies that used amplitude of low-frequency fluctuation (ALFF) and 11 studies that used regional homogeneity (ReHo). All ALFF and ReHo studies compared PD patients with healthy controls. We also performed a validation RS-fMRI study of ALFF and ReHo in a frequency-dependent manner for a novel dataset consisting of 49 PD and 49 healthy controls.

**Findings:**

Decreased ALFF was found in the left putamen in PD by meta-analysis. This finding was replicated in our independent validation dataset in the 0.027–0.073 Hz band but not in the conventional frequency band of 0.01–0.08 Hz.

**Conclusions:**

Findings from the current study suggested that decreased ALFF in the putamen of PD patients is the most consistent finding. RS-fMRI is a promising technique for the precise localization of abnormal spontaneous activity in PD. However, more frequency-dependent studies using the same analytical methods are needed to replicate these results.

Trial registration: NCT NCT03439163. Registered 20 February 2018, retrospectively registered.

## Background

Parkinson's disease (PD) is a progressive neurological degenerative disorder that is characterized by bradykinesia, rigidity, tremor, and postural instability. The main pathological feature of PD is the progressive loss of dopamine neurons in the substantia nigra and putamen [1]. However, it remains unclear how these pathological changes lead to parkinsonian symptoms. To answer this question, many neuroimaging studies that used functional magnetic resonance imaging (fMRI) have investigated PD-related neural abnormalities and found that PD patients showed abnormal activity in the striatum and brain stem, as well as in other brain regions [2]. Most fMRI studies have been focused on motor-related neural changes in PD. Herz et al. conducted a coordinate-based (CB) meta-analysis on motor task-related functional neuroimaging studies [3] and found a consistently decreased activation in the posterior portion of the putamen in PD patients during motor tasks.

While task-related fMRI holds the advantage of being able to assess specific activation corresponding to specific tasks, different tasks activate different brain regions and hence the results are less suitable for meta-analysis. Resting-state (RS) fMRI has two intrinsic advantages: it is noninvasive and task free. Task-free RS is a simpler experimental design for PD investigation. Therefore, RS-fMRI design is very similar across studies, and is more suitable for meta-analysis. There have been three published CB meta-analysis investigations of RS-fMRI studies in PD [4-6]. The CB meta-analysis study by Tahmasian and colleagues included 28 publications, in which a variety of RS-fMRI analytical methods were used, such as amplitude of low-frequency fluctuation (ALFF) or its derivative, fractional ALFF (fALFF); regional homogeneity (ReHo); and various network analytical methods [6]. In contrast, the analytical methods in the original studies that were included in the two CB meta-analysis studies by Pan and colleagues were very similar, i.e., ReHo [4] and ALFF/fALFF [5], respectively. In between-group comparison studies, ALFF/fALFF and ReHo are usually used in “voxel-wise whole-brain” analysis with very similar preprocessing parameters across studies. Therefore, these studies are suitable for inclusion in meta-analysis [7]. In the CB meta-analysis studies by Pan and colleagues, the authors found decreased ReHo [4] and decreased ALFF [5] in the putamen, which was consistent with the hypothesis of decreased dopaminergic function in the putamen [1].

Frequency-dependent or subfrequency band analysis is drawing more and more attention in RS-fMRI studies since the work by Zuo and colleagues [8]. They observed a higher RS-fMRI fALFF at a frequency band of 0.027–0.073 Hz (namely, slow-4) than that at 0.01–0.027 Hz (namely, slow-5) in the basal ganglia, which is a critical subcortical area in PD pathology. However, only two of the previous ALFF or ReHo PD studies investigated subfrequency bands. Hou and colleagues found similar decreased ALFF in the putamen for the two subfrequency bands [2], but Zhang and colleagues did not find abnormal ALFF in the putamen in any of the two frequency bands [9]. Although higher-frequency band (>0.1 Hz) of the RS-fMRI signal could be contaminated by physiological noise, including respiratory noise (around 0.3 Hz) and heart beats (around 1.2 Hz), quite a few studies found that RS-fMRI signal at higher frequency bands was of pathophysiological [10, 11] and physiological [12] significance. It would be interesting to perform more studies at subfrequency bands in RS-fMRI studies on PD.

In the current study, we carried out a CB meta-analysis on previous PD resting-state fMRI studies using ALFF/fALFF and ReHo separately, similar to previous meta-analysis [4, 5]. However, we also added five articles that were published after the CB meta-analysis articles by Pan and colleagues [4, 5]. Furthermore, to validate the results of our CB meta-analysis, we analyzed ALFF and ReHo in the conventional frequency band, as well as in subfrequency bands, and we studied an independent and relatively large cohort of PD patients and healthy controls.

It should be noted that both ALFF and ReHo are metrics for measuring local activity. Both methods have been widely used in studies on brain disorders in whole-brain voxel-wise analysis. A study showed that ReHo and ALFF could reveal convergent abnormal local activity in some brain areas [13], but ReHo and ALFF also detected different brain regions with abnormal brain activity. The two methods are very different mathematically. ReHo depicts the local synchronization of the time course of neighboring voxels, while ALFF depicts the fluctuation amplitude of every signal time course. Hence, both separate and combined analyses are necessary. Therefore, in addition to separate meta-analysis on ALFF and ReHo, we were also interested in combining both ALFF and ReHo studies into our meta-analysis. Furthermore, we investigated the frequency-specific features of PD-related brain activity in an independent dataset to improve our understanding of the neurophysiological changes underlying PD.

## Data Description

The data for the current study included two parts. Part 1 was for a CB meta-analysis including coordinates, *t* value, and sample size. These data were extracted from published articles that used RS-fMRI in PD. Part 2 was for validation purposes and used RS-fMRI data from 80 patients with PD and 52 healthy participants. After quality control (see details in Analyses section), 49 patients with PD and 49 healthy participants were entered into the final analysis. All data in the current study can be used for further validation and exploratory studies.

## Analyses

### Literature search

On 28 June 2017, we conducted a PubMed literature search [14] using the search strings as follows: ((Parkinson[Title/Abstract]) OR (Parkinson's[Title/Abstract])) AND ((“resting-state fMRI” OR ALFF OR ReHo OR “default mode network”)). A total of 138 articles were retrieved.

We also reviewed articles and references to retrieve additional articles. An additional study, using the Kendall coefficient of concordance (KCC) method, was included in the current meta-analysis after carefully reviewing a recent publication of meta-analysis [4] because KCC is the same algorithm as that of ReHo [15]. Only RS-fMRI studies written in English were eligible. The inclusion criteria were as follows: articles reporting original data; analyses using ReHo or ALFF/fALFF and based on the whole brain; articles reporting results on RS data in adult PD patients and studies aimed at comparing PD with healthy controls; studies reporting results with coordinates from group comparisons (PD vs healthy controls) in Montreal Neurological Institute (MNI) or Talairach space; patients were in an off-state; and patients were pretreatment, presurgery, and noncomorbid. According to these criteria, two investigators (J.W., H.-x.W.) independently searched and selected the articles from PubMed. Final decisions were made by a neurologist (T.W.); 26 RS-fMRI studies (15 studies using ALFF/fALFF [2, 9, 16-28] and 11 studies using ReHo [29-39]) were included in the present CB meta-analysis (Table 1).

**Table 1:** Original articles included in the present meta-analysis study

References	Indices	Sample size (female)	Age (SD)	Disease duration	H&Y (SD)	UPDRS III (SD)	Foci	FWHM	Scanner	Comparison	Medication status
Hou et al., 2014	ALFF	PD 101 (42)	59.84 (7.15)	7.23 (4.42)	1.87 (0.71)	25.54 (11.51)	7	3	3 T	PD OFF vs HC slow-4 (0.027–0.073 Hz) ^#^	Off-state
		HC 102 (42)	59.91 (7.09)				4			PD OFF vs HC slow-5 (0.01–0.027 Hz) ^#^	
							7			HC vs PD OFF slow-4 (0.027–0.073 Hz) ^#^	
							5			HC vs PD OFF slow-5 (0.01–0.027 Hz) ^#^	
Kwak et al., 2012	ALFF	PD 24 (2)	64.3 (8)	5.4 (3)	2.2 (0.3)	18.5 (8)*	4	8	3 T	PD OFF vs HC	Off-state
		HC 24 (5)	63.3 (7)				4			HC vs PD OFF	
	fALFF						6			PD OFF vs HC	
							5			HC vs PD OFF	
Wen et al., 2013	ALFF	PD 16 (8)	60.7 (18.7)	5.6 (7.4)	1.5 (1)	33.8 (24.2)	11	5	3 T	PD-NDep OFF vs HC	Off-state
		HC 21 (8)	55.4 (16.4)				8			HC vs PD-NDep OFF	
Yao et al., 2015	ALFF	PD 12 (8)	63.4 (7.4)	8.4 (5.1)	2.8 (0.9)	18.0 (12.9)	5	4	3 T	PDnonVH vs HC	N/A
		HC 14 (8)	64.1 (4.0)				2			HC vs PDnonVH	
Zhang et al., 2013	ALFF	PD 82 (47)	59.7 (11.9)	7.05 (6.01)	N/A	20.24 (8.44)	7	8	3 T	PD OFF vs HC slow-5 (0.01–0.027 Hz) ^#^	Off-state
		HC 77 (46)	58.6 (8.5)				5			HC vs PD OFF slow-4 (0.027–0.073 Hz) ^#^	
							6			HC vs PD OFF Slow-5 (0.01–0.027 Hz) ^#^	
Luo et al., 2014	ALFF	PD 30 (15)	53.64 (10.18)	2.12 (1.3)	2	26.83 (12.44)	1	8	3 T	PD-NDep OFF vs HC	Off-state
		HC 30 (15)	51.9 (7.7)								
Chen et al., 2015	ALFF	PD 19 (7)	64.8 (8.34)	6.68 (4.85)	2.13 (0.984)	21.6 (11.6)	5	8	3 T	PIGD vs HC	N/A
		HC 22 (10)	65.1 (5.0)				5			HC vs PIGD	
Skidmore et al., 2013	ALFF	PD 14 (3)	62 (9)	N/A	N/A	37 (13)	1	6	3 T	PD vs HC	Off-state
		HC 15 (6)	65 (13)				7			HC vs PD	
Hu et al., 2015	fALFF	PD 17 (7)	60.29 (12.03)	3.94 (2.57)	N/A	17.11 (6.12)	3	8	3 T	PD vs HC	N/A
		HC 20 (9)	58.48 (6.89)								
Gao et al., 2016	ALFF	PD 16 (6)	64.13 (6.71)	5.69 (4.07)	1.73 (0.57)	16.93 (3.86)	18	8	3 T	HC vs PD cognitively normal	Off-state
		HC 16 (7)	63.5 (6.49)								
Li et al., 2016	ALFF	PD 16 (10)	62.8 (6.6)	4 (4.3)	2.2 (0.8)	22.1 (12.5)	1	6	3 T	HC vs PD-nRBD	Off-state
		HC 19 (8)	62.7 (8.1)								
Xiang et al., 2016	ALFF	PD 24 (12)	62.7 (7.4)	7.0 (3.3)	2.2 (0.9)	22.0 (7.0)	3	6	3 T	HC vs PD OFF	Off-state
		HC 22 (11)	65.6 (6.9)				4			PD OFF vs HC	
Zhang et al., 2016	ALFF	PD 32 (10)	65 (8.38)	4.04 (3.98)	2.18 (0.67)	21.6 (9.99)	4	6	3 T	PD-NF vs HC	Off-state
		HC 25 (13)	64.6 (4.49)								
Tang et al., 2017	ALFF/fALFF	PD 51 (24)	53.2 (11)	5.745 (5.026)	2.353 (0.764)	48.59 (23.41)	3	8	3 T	PD vs HC	Off-state
		HC 50 (29)	51.5 (10.7)								
Possin et al., 2013	fALFF	PD 12 (9)	73.9 (5.9)	9 (7)	N/A	30.8 (14.5)	48	4	3 T	PD vs HC	Off-state
		HC 12 (11)	72.9 (5.2)								
Choe et al., 2013	ReHo	PD 22 (12)	58.3 (2.4)	3.2 (0.4)	1.6 (0.2)	10.4 ± 1.2	2	9	3 T	PD OFF vs HC	Off-state
		HC 25 (15)	58.3 (1.7)				1			HC vs PD OFF	
Wu et al., 2009	ReHo	PD 22 (6)	59.5 (8.1)	4.1 (1.8)	1.7 (0.5)	25.6 (8.1)	11	4	1.5 T	PD OFF vs HC	Off-state
		HC 22 (6)	59.7 (N/A)				13			HC vs PD OFF	
Yang et al., 2013	ReHo	PD 17 (7)	60.43 (9.65)	1.6 (1.06)	1.2 (0.33)	20.57 (3.82)	10	4	1.5 T	PD medication-naive vs HC	Off-state
		HC 17 (7)	60.73 (8.57)				7			HC vs PD medication-naive	
Sheng et al., 2014	ReHo	PD 21 (7)	57.3 (6.1)	4.0 (2.4)	1.95 (0.63)	43.8 (8.2)	3	4	3 T	nD-PD OFF vs HC	Off-state
		HC 25 (9)	56.7 (5.3)								
Jiang et al., 2015	ReHo	PD 13 (6)	68.46 (6.5)	2.83 (2.38)	2.5 (0.46)	19.31 (8.33)	11	4	3 T	PIGD vs HC	Off-state
		HC 17 (8)	63.71 (5.21)				16			HC vs PIGD	
Li et al., 2016	ReHo	PD 23 (12)	63 (7.1)	7 (3.3)	2.2 (0.9)	38 (18.6)*	10	6	3 T	PD vs HC	Off-state
		HC 20 (9)	65.3 (7.0)				4			HC vs PD	
Zhang et al., 2015	ReHo	PD 27 (11)	63.38 (9.46)	4.17 (4.07)	2.21 (0.67)	19.88 (6.7)	13	4	3 T	PD-AR vs HC	Off-state
		HC 26 (15)	59.31 (7.15)				8			HC vs PD-AR	
Sheng et al., 2016	ReHo	EOPD 18 (8)	45.4 (6.07)	3.04 (1.99)	2.03 (0.78)	16.94 (5.07)	1	4	3 T	Young HC vs EOPD	Off-state
		Young HC 19 (10)	45.8 (3.55)				1			EPOD vs Young HC	
		LOPD 21 (9)	63.6 (4.84)	3.1 (1.78)	2.0 (0.62)	18.61 (4.51)	2			Old HC vs LOPD	Off-state
		Old HC 18 (10)	61.7 (9.73)								
Wen et al., 2016	ReHo	rPD 12 (4)	60.8 (7.02)	5 (N/A)	N/A	28.9 (10.9)	7	4	3 T	rPDpre vs HC	Off-state
		HC 31 (16)	59.6 (7.65)				2			HC vs rPDpre	
		lPD 14 (8)	61.4 (7077)	5.5 ((N/A)	N/A	26.4 (15.6)	6			lPDpre vs HC	Off-state
							8			HC vs lPDpre	
Yeo et al., 2012	ReHo	PD 12 (6)	53.5 (10.9)	2.67 (2.3)	1.5 (0.6)	7.8 (3.9)	8	9	3 T	HC vs PD before stimulations	Off-state
		HC 12 (6)	55.9 (9.8)								
Borroni et al., 2015	ReHo	PD 11 (1)	66.3 (3.8)	7.8 (3.1)	N/A	10.7 (5.4)	3	8	1.5 T	HC vs PD	N/A
		HC 10 (7)	62.2 (8.0)								

In two studies (Hou et al., 2014; Zhang et al., 2013), subfrequency analysis was performed. The two subfrequency bands were taken into one text file for meta-analysis.

*The score is uncertain whether full UPDRS or part III because we failed to contact the authors.

Abbreviations: ALFF: amplitude of low-frequency fluctuation; EOPD: early onset PD; fALFF: fractional amplitude of low-frequency fluctuation; FWHM: full-width at half maximum; HC: healthy control; H & Y: Hoehn and Yahr scale; lPDpre: pre-left-side-thalamotomy PD; LOPD: late onset PD; nD-PD: nondepressed PD; N-VH PD: no visual hallucinations PD; PD: Parkinson's disease; PD-AR: akinetic-rigid PD; PD-nRBD: no rapid eye movement sleep behavior disorder PD; PD-NDep: nondepressed PD; PD OFF: PD off medication; PIGD: postural instability gait difficulty PD; ReHo: regional homogeneity; rPDpre: pre-right-side-thalamotomy PD; UPDRS: unified Parkinson's disease rating scale.

### Meta-analysis of ALFF and ReHo studies

Signed differential mapping (SDM) software (version 5.141 for Windows) [40] was used for meta-analysis. One feature of SDM is “the representation of both positive differences and negative differences in the same map, thus obtaining a signed differential map (“SDM”).” Another feature is “the use of effect sizes (leading to effect-size SDM or “ES-SDM”)^40^].” Two directions of abnormality were probed: RS activity increases and decreases in PD patients compared with healthy controls. ALFF measures the fluctuation amplitude of the low-frequency band (usually 0.01–0.08 Hz) of a single time course [41], and fALFF is the ratio of the ALFF to the fluctuation amplitude of the full-frequency band [42]. ReHo measures the local synchronization of the time courses of the nearest neighboring voxels (usually 27 voxels) [15]. Although ALFF and ReHo showed significant correlation in most voxels [43], a previous comparison study showed that ALFF and ReHo could detect different abnormal brain areas [13]. Therefore, similar to two previous CB meta-analysis articles [4, 5], we first performed meta-analysis separately on ALFF/fALFF studies (15 studies) and ReHo studies (11 studies, including 13 comparisons) (Table 1) and then combined ALFF and ReHo (see below). The analysis procedure included listing the peak coordinates and *t*-values from each study; using the files prepared in i) to recreate the effect-size maps (standard stereotactic space) of the original studies with 10 Monte Carlo randomizations and full-width at half maximum (FWHM) 20 mm; and generating the mean map in a voxel-wise manner weighted by the sample size, variance, and between-study heterogeneity. A combined threshold of *P* < 0.001 (uncorrected for false discovery rate [FDR]) with peak height Z value >1 was adopted as recommended in the SDM, together with extent threshold >10 voxels [44, 45].

In addition to the above separate meta-analysis on ALFF and ReHo as by Pan and colleagues [4, 5], we further performed a combined meta-analysis of all ALFF and ReHo studies (15 ALFF and 13 ReHo = 28 comparisons; Table 1). Results were also thresholded at *P* < 0.001, uncorrected for FDR, with a peak height Z value >1 and extent threshold >10 voxels [44, 45].

### Validation study of ALFF and ReHo on an independent dataset of RS-fMRI

#### Participants

This validation study contains RS-fMRI data of 80 patients with PD and 52 healthy participants. After head motion control, 12 patients were excluded, and matching for age and gender was conducted. A total of 98 right-handed participants, made up of 49 PD patients (26 females) and 49 age- and gender-matched healthy controls (26 females) were enrolled in the final analysis (mean age ± standard deviation: 62.3 y ± 8.0, 61.8 y ± 8.3, respectively; Table 2). The PD diagnoses were based on the UK Parkinson's Disease Society Brain Bank Clinical Diagnostic Criteria [46]. Patients were assessed using the unified Parkinson's disease rating scale III [47] and the Hoehn and Yahr disability scale [48]. Exclusion criteria included history of head trauma, substance abuse, or psychiatric disorder. For healthy controls, additional exclusion criteria included any history of neuropsychiatric disorders. The present investigation was performed according to the Declaration of Helsinki and was approved by the Medical Research Ethics Committee at Xuanwu Hospital, Capital Medical University. All participants gave written informed consent prior to participation.

**Table 2:** Demographic characteristics

	Parkinson's disease	Control
Male/Female	N = 49 (23/26)	N = 49 (23/26)
Age (y)	62.3 ± 8.0	61.8 ± 8.3
Disease duration (y)	5.5 ± 3.8	-
Disease stage (Hoehn and Yahr scale)	1.9 ± 0.7	-
Unified Parkinson's disease rating scale III	23.3 ± 11.0	-
Mini-mental State Examination	28.3 ± 1.6	-

### Data acquisition

fMRI data were acquired on a 3T MR scanner (Trio system; Siemens Magnetom scanner, Erlangen, Germany) with gradient-echo echo-plannar imaging sequences. Whole brain fMRI scanning with three slightly different parameters was carried out (see Table 3). All participants were instructed to keep their eyes closed, relax, remain motionless, not think of anything in particular, and not fall asleep. Foam pads were used to minimize head motion.

**Table 3:** Parameters of the three resting-state fMRI datasets

	Dataset 1	Dataset 2	Dataset 3
Time of repetition (ms)	2,000	2,000	2,000
Time of echo (ms)	30	40	40
Field of view	220 mm × 220 mm	256 mm × 256 mm	256 mm × 256 mm
Matrix	64 × 64	64 × 64	64 × 64
Flip angle	90	90	90
Slice thickness	3	4	4
Gap	0.5	1	1
Slices	32	28	28
Volumes	180	239	300

### Data analyses

The minimum time points were 180 (dataset 1, Table 3). The extra time points of datasets 2 and 3 were discarded and hence 180 time points were left. The RS-fMRI data preprocessing included the following steps: 1) discarding the first 10 volumes to allow the signal to reach equilibrium and the subjects to adapt to the circumstances; 2) correcting for the acquisition time delay between slices; 3) rigid-body realigning for estimation and correction of the motion displacement (participants whose head motion exceeded 2 mm in translation or 2 degrees in rotation in any direction were excluded); 4) normalizing to MNI space using the echo-plannar imaging template in statistical parametric mapping 8 [49]; 5) regressing out of the six motion parameters; 6) removing the linear trend; and 7) band-pass filtering for five frequency bands (0–0.01, 0.01–0.027, 0.027–0.073, 0.073–0.198, and 0.198–0.25 Hz, as well as 0.01–0.08 Hz). Most of the previous PD studies investigating amplitude of low-frequency fluctuation used ALFF but not fALFF. We therefore analyzed ALFF only. For calculating ALFF, data were further smoothed, with a Gaussian kernel of 6 mm FWHM.ALFF was then calculated using the REST toolkit [50]. For ReHo, the calculation was performed first, and then the 6 mm FWHM smoothing was carried out on the ReHo maps.

### Statistical analyses

Two-sample *t* tests were performed to explore the differences between the two groups in a voxel-wise manner for ALFF and ReHo, respectively, for each subfrequency band as well for the conventional frequency band (0.01–0.08 Hz). Monte Carlo simulation (AlphaSim) was applied for the multiple comparison correction within a whole brain mask by using DPABI_V3.0 [51] (DPABI, RRID:SCR_010501) software [52]. DPABI estimates the smoothness of each statistic map and hence yields effective kernel size of smoothness for each map. Then, the smoothness was used for the correction. It is believed that simply taking the size of the Gaussian kernel that was applied during preprocessing to AlphaSim is incorrect [53]. DPABI prevents that sort of error by estimated effective smoothness. Although Monte Carlo simulations in DPABI are based on AFNI's 3dClustSim, the specific algorithm used is not by the bug reported in Eklund et al., 2016 since version 1.2_141101 [54, 55]. The corresponding estimated smoothness and minimal cluster size are listed in Table 4 for each frequency band of ALFF and ReHo. The voxel-level *P* value was set at <0.001 as recommended [54]. The corrected *P* value was <0.05.

**Table 4:** Estimated smoothness and cluster size of T maps (PD vs healthy controls based on validation study)

	Estimated smoothness (mm)			
Frequency band, Hz	FWHM x	FWHM y	FWHM z	Cluster size (number of voxels)
**ALFF**				
0.01–0.08	10.42	11.20	10.68	46
0–0.01	8.81	9.23	9.03	30
0.01–0.027	8.80	9.15	9.04	31
0.027–0.073	9.53	9.86	9.79	35
0.073–0.198	7.87	8.22	7.82	23
0.198–0.25	7.41	7.61	7.04	19
**ReHo**				
0.01–0.08	12.24	13.03	13.37	64
0–0.01	13.07	14.35	13.80	74
0.01–0.027	12.34	12.55	12.68	62
0.027–0.073	12.39	13.12	13.49	69
0.073–0.198	12.07	13.54	12.93	65
0.198–0.25	11.53	12.18	11.84	56

### Findings

#### Meta-analysis on ALFF and ReHo studies

**Figure 1:**
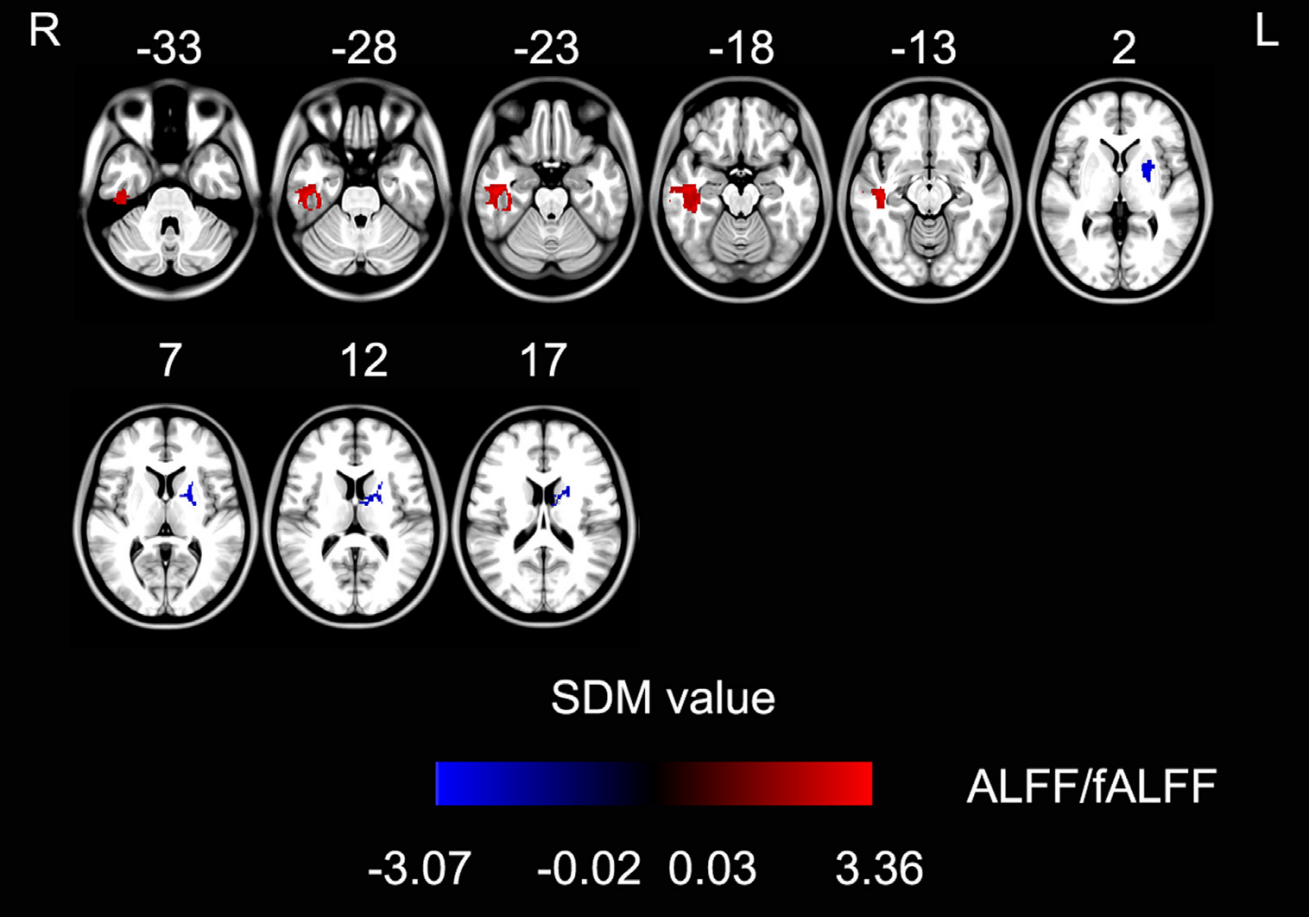
ALFF/fALFF differences between Parkinson's disease patients and healthy controls in coordinate-based meta-analysis (*P* < 0.001, uncorrected for false discovery rate; peak height *Z* value > 1; extent threshold >10 voxels). The warm color represents an increased SDM value and the cold color represents a decreased SDM value in Parkinson's disease patients. Abbreviations: ALFF: amplitude of low-frequency fluctuation; fALFF: fractional amplitude of low-frequency fluctuation; SDM: signed differential mapping.

In the meta-analysis of SDM for ALFF, an increased ALFF in PD patients compared with controls was found in the right inferior temporal gyrus. A decreased ALFF in PD patients compared with controls was found in the left pallidum/putamen and the right cuneus cortex (Fig. 1, Table 5).

**Table 5:** Brain regions showing differences between PD and healthy controls based on meta-analysis

Brain region	Brodmann area	Montreal Neurological Institute (X Y Z)			SDM *Z* value	Cluster size (mm^3^)	*P* value
**SDM (15 ALFF/fALFF studies)**							
**PD > Controls**							
Right inferior longitudinal fascicules		42	−28	−14	3.37	6,256	0.000000894
**PD < Controls**							
Left pallidum/putamen		−22	4	6	3.08	2,160	0.000015318
Right cuneus cortex	19	6	−88	26	2.39	80	0.000558496
**SDM (11 ReHo studies)**							
**PD > Controls**							
Left inferior parietal lobule	39	−44	−66	38	2.55	4,552	0.000009179
Right superior frontal gyrus/pre-supplementary motor area	9	10	38	48	2.24	2,120	0.0000844
Right inferior parietal lobule	40	56	−42	40	1.95	360	0.000498116
**PD < Controls**							
Right putamen/insula	48	36	−4	4	2.46	8,744	0.000002623
Right precentral gyrus	6	44	−4	48	1.78	312	0.000464916
**SDM (15 ALFF/fALFF studies + 11 ReHo studies)**							
**PD > Controls**							
Right inferior longitudinal fascicules		44	−30	−16	3.04	2,168	0.000040650
Brain stem		2	−28	−26	2.58	224	0.000389218
**PD < Controls**							
Left pallidum/putamen		−22	10	12	2.92	2,504	0.000040233
Right insula	47	30	24	−2	2.51	248	0.000307441

Abbreviations: ALFF: amplitude of low frequency fluctuation; fALFF: fractional amplitude of low frequency fluctuation; PD: Parkinson's disease; ReHo: regional homogeneity; SDM: signed differential mapping.

**Figure 2:**
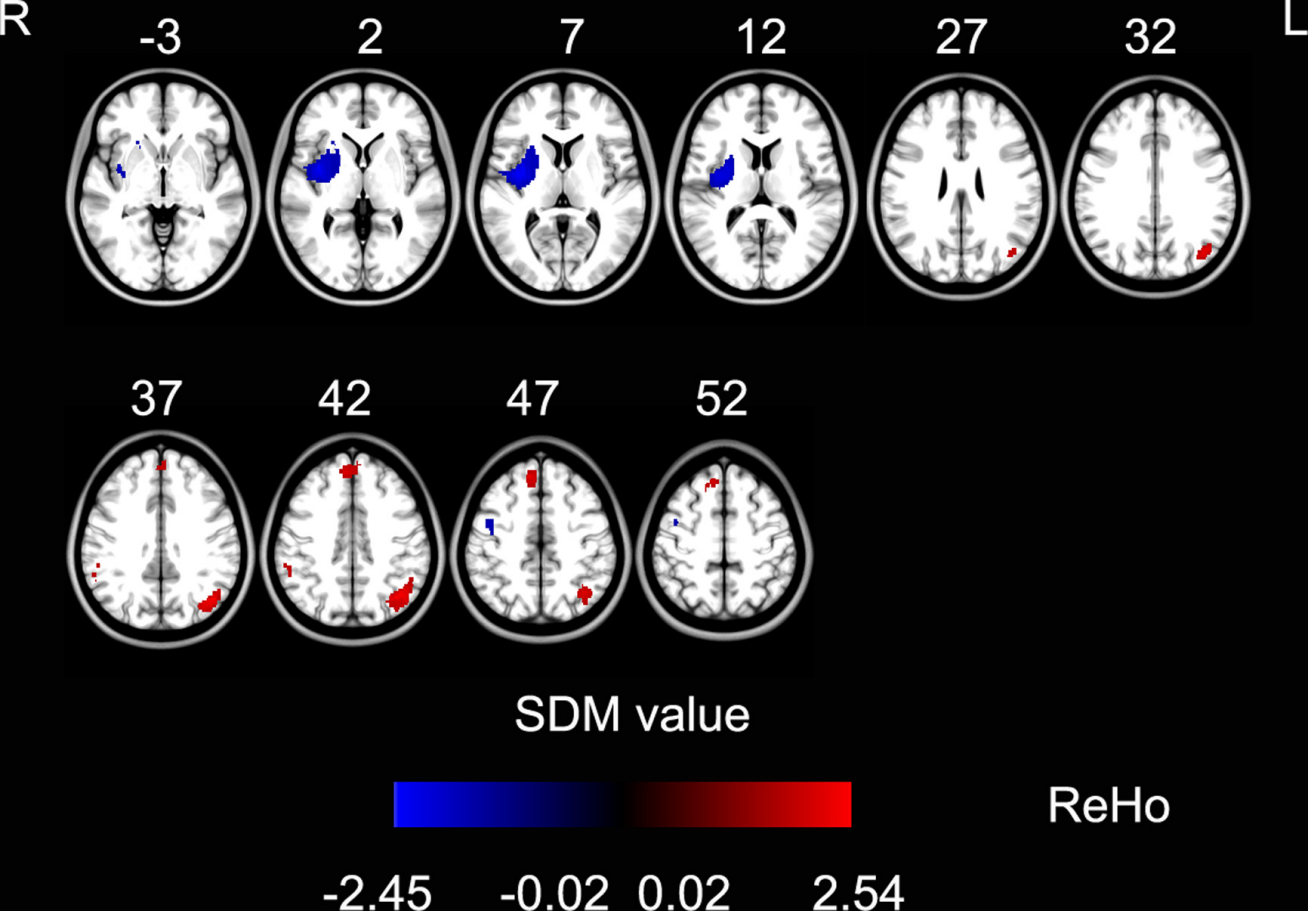
ReHo differences between Parkinson's disease patients and healthy controls in coordinate-based meta-analysis (*P* < 0.001, uncorrected for alse discovery rate; peak height *Z* value > 1; extent threshold >10 voxels). The warm color represents an increased SDM value and the cold color represents a decreased SDM value in Parkinson's disease patients. ReHo: regional homogeneity; SDM: signed differential mapping.

Meanwhile, using SDM for ReHo, increased ReHo was observed in the bilateral inferior parietal lobule and the right superior frontal gyrus/pre-supplementary motor area (SMA) (Brodmann area [BA] 9) in PD patients. In addition, decreased ReHo was observed in the right putamen and right precentral gyrus (BA 6) in PD patients (Fig. 2, Table 5).

**Figure 3:**
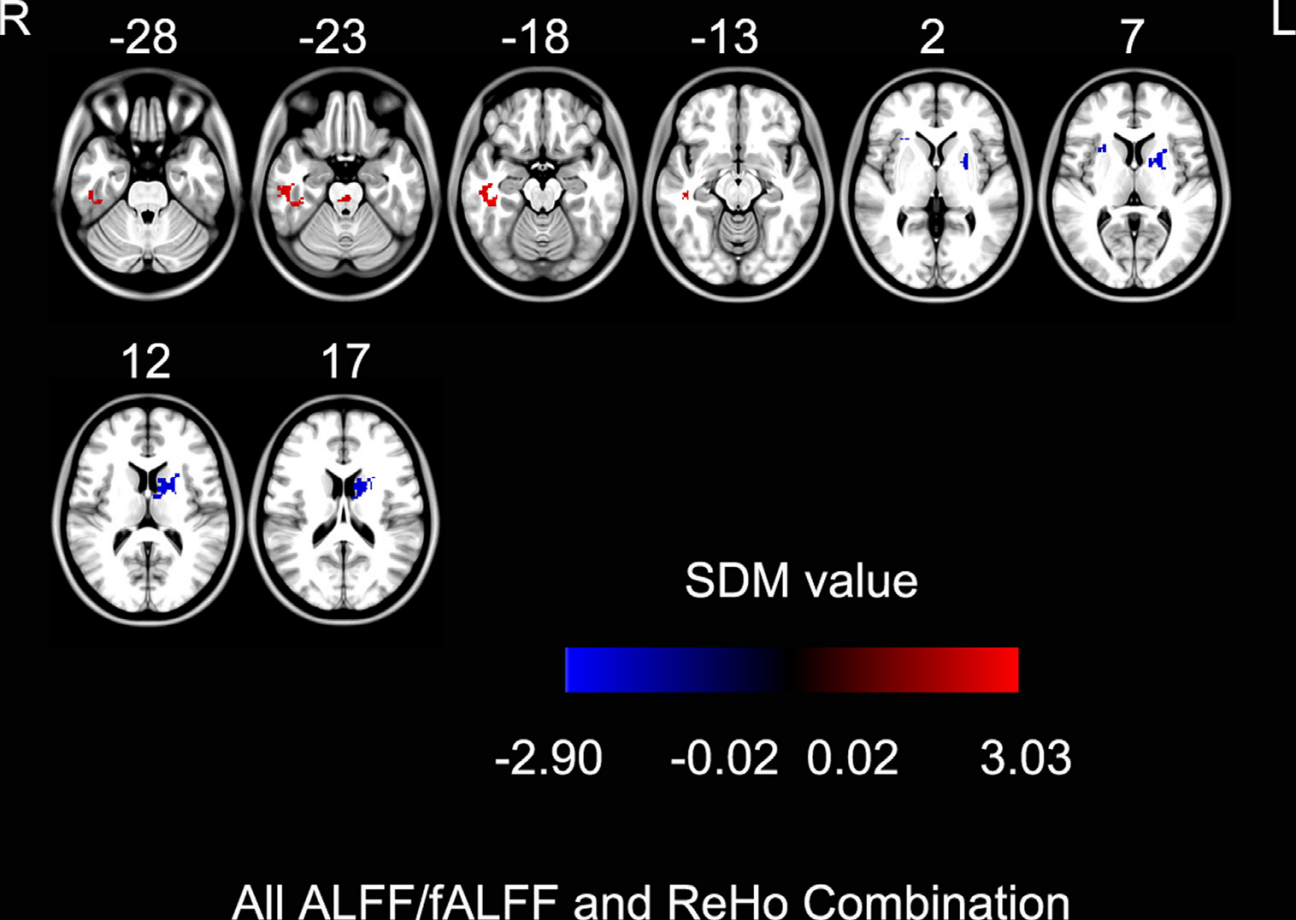
Combined ALFF and ReHo differences between Parkinson's disease patients and healthy controls in coordinate-based meta-analysis (*P* < 0.001, uncorrected for false discovery rate; peak height *Z* value > 1; extent threshold >10 voxels). The warm color represents an increased SDM value and the cold color represents a decreased SDM value in Parkinson's disease patients. ALFF: amplitude of low-frequency fluctuation; fALFF: fractional amplitude of low-frequency fluctuation; ReHo: regional homogeneity; SDM: signed differential mapping.

In the meta-analysis of SDM for combination of ALFF and ReHo, increased spontaneous brain activities in PD patients compared with controls were found in the right inferior temporal gyrus and right brain stem. Decreased spontaneous brain activities were found in the left pallidum/putamen and the right insula (BA 47) (Fig. 3, Table 5).

#### Results of the validation study of ALFF and ReHo

**Figure 4:**
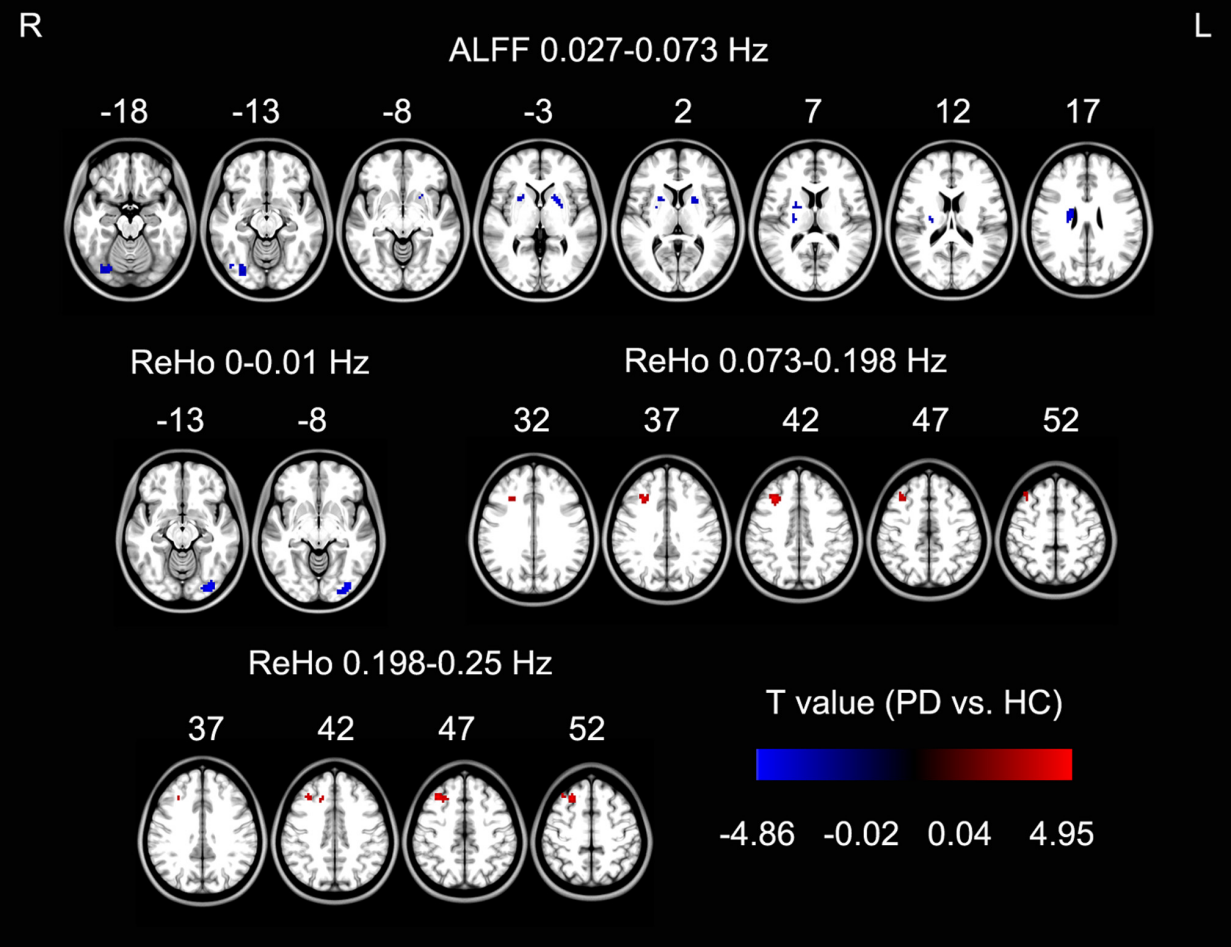
ALFF/ReHo differences between PD and HC in the validation study. The warm color represents an increased ALFF/ReHo and the cold color represents a decreased ALFF/ReHo in PD (voxel-level *P* < *0.001*, corrected *P* < 0.05). Abbreviations: ALFF: amplitude of low-frequency fluctuation; ReHo: regional homogeneity; PD: Parkinson's disease; HC: healthy controls.

Compared with healthy controls, PD patients had decreased ALFF in the bilateral putamen and right fusiform at 0.027–0.073 Hz (Fig. 4, Table 6). PD patients also had decreased ReHo in the left inferior occipital gyrus at 0–0.01 Hz and increased ReHo in the right middle frontal gyrus at 0.073–0.198 Hz and 0.198–0.25 Hz (Fig. 4, Table 6). The other frequency bands, including conventional 0.01–0.08 Hz, did not show significant differences of ALFF or ReHo. Among these brain regions, the decreased ALFF at 0.027–0.073 Hz in the left putamen was overlapped with our findings of decreased ALFF in the meta-analysis.

**Table 6:** Brain regions of validation study showing differences between PD patients and healthy controls

Brain region	Frequency band (Hz)	Brodmann area	Montreal Neurological Institute (X Y Z)			Peak *t* value	Cluster size (mm^3^)	*P* value
**ALFF**								
**PD < Controls**								
Right putamen	0.027–0.073		24	12	6	4.22	1,296	<0.001
Left putamen	0.027–0.073		−21	9	3	4.90	1,188	<0.001
Right fusiform	0.027–0.073	19	30	−75	−15	4.53	1,512	<0.001
White matter			21	−9	27	4.79	1,512	<0.001
**ReHo**								
**PD < Controls**								
Left inferior occipital gyrus	0–0.01	19	−33	−87	−12	4.37	2,322	<0.001
**PD > Controls**								
Right middle frontal gyrus	0.073–0.198	46	30	21	39	4.97	2,187	<0.001
Right middle frontal gyrus	0.198–0.25	9	33	27	48	4.83	1,782	<0.001

Abbreviations: ALFF: amplitude of low-frequency fluctuation; PD: Parkinson's disease; ReHo: regional homogeneity.

## Discussion

Using the SDM meta-analysis, we detected some characteristic PD-related neural changes in the resting state. For example, there was altered local activity in the putamen and the pre-SMA. A finding of decreased ALFF in the left putamen from our validation study was very consistent with the results of our CB meta-analysis, which was also consistent with findings from a previous CB meta-analysis [5]. The increased ReHo in the validation dataset was not consistent with our CB meta-analysis results and the previous one [4].

### Methodology: whole-brain voxel-wise comparison and image-based meta-analysis

Compared with positron emission tomography, RS-fMRI has the advantages of lower cost, better temporal resolution, and no ionizing radiation. Since 2009 when the first PD RS-fMRI article was published [30], approximately 150 research articles have described RS-fMRI in PD. To ensure that the analytical methods were as similar as possible, two previous CB meta-analysis articles included only ALFF [5] or ReHo [4], because many other analytical methods are not “whole-brain voxel-wise” and hence not suitable to CB meta-analysis. For example, the “seed” location of seed-based functional connectivity varies greatly across studies, and for independent component analysis, researchers may be interested in any network or component. Likewise, a very small portion of the existing graph theory studies are whole-brain voxel-wise [56]. Instead, most of them are region based due to the computational cost of this method. Unfortunately, because only a small portion of RS-fMRI studies have used whole-brain voxel-wise analytic methods, the two previous CB meta-analysis studies have included only a limited number (10 or fewer) of articles in which the same analytical methods were used. After our careful screening of PD RS-fMRI articles, we found a few additional eligible studies that used whole-brain voxel-wise analytic methods, i.e., ALFF/fALFF (15 papers) and ReHo (11 article; Table 1). It has been suggested that for meta-analysis, unthresholded effect size maps (named image-based) were better than coordinate-based meta-analysis [57]. Therefore, effect size maps from whole-brain voxel-wise comparison should be widely performed in future RS-fMRI studies.

### Methodology: ALFF vs ReHo

ALFF measures the amplitude of fluctuation of every single voxel, while ReHo measures the local synchronization of the nearest neighboring voxels. The two metrics are the two most widely used methods for depicting local activity [58]. Also, it has been shown that ALFF and ReHo were among the RS-fMRI metrics that have the highest test–retest reliability [59]. Although the ALFF and ReHo methods are mathematically different, both methods measure the local activity of spontaneous brain activity. A previous study compared the two methods in attention deficit hyperactivity disorder [13] and found a few convergent abnormal regions for ALFF and ReHo, albeit some divergent abnormal activity already existed. Therefore, we performed a combined meta-analysis on all ALFF and ReHo studies. It was shown that the results of combined meta-analysis of all ALFF and ReHo studies looked like complimentary results of separate meta-analysis mutually between ALFF and ReHo. An interesting result is the decreased spontaneous activity in the bilateral putamen. These results were replicated in the independent validation dataset. Comparing the results of ALFF and ReHo either by meta-analysis or independent validation analysis, almost no convergent results were found for the two metrics. More studies are needed in the future to compare the two methods.

### Consistent decreased ALFF in the left putamen

There was decreased ALFF in the left putamen in PD patients in both our validation dataset and CB meta-analysis investigation. These findings align with previous findings that the striatal dysfunction in PD has been consistently reported in previous studies [60-67]. It has been established that dopamine uptake is reduced in the putamen in PD [68], which is a critical factor that leads to major parkinsonian symptoms. Cells loss from the substantia nigra in PD results in dopamine expending in the striatum, with putamen being affected [69, 70]. It has been reported that ^18^F-dopa uptake in putamen in PD is associated with the clinical severity of locomotor disability, and ^18^F-dopa reduction in putamen is associated is associated with the degree of rigidity and bradykinesia [68, 71]. A recent meta-analysis of motor-related task fMRI studies also found decreased activity in the putamen in PD patients [3]. Notably, in our validation dataset, we found that the decreased ALFF was mainly found in a subfrequency band of 0.027–0.073 Hz, namely, slow-4 [8]. Either ALFF or ReHo abnormality in the left putamen was not found in the conventional frequency band of 0.01–0.08 Hz. We suggest that future RS-fMRI studies pay close attention ton subfrequency analysis in order to validate this finding. Further, the physiological importance of each subfrequency band of RS-fMRI should also be investigated.

### Inconsistent ReHo and ALFF findings

There was decreased ReHo in the right putamen in PD patients in both our CB meta-analysis and a previous CB meta-analysis [4]. However, no ReHo changes were found in the validation dataset (Fig. 4). This discrepancy in findings between ALFF and ReHo might be explained by the differences in the two methods as discussed in the Methodology: ALFF vs ReHo section. Further, except for the consistent results in the left putamen in ALFF, most meta-analysis results were not reproducible in the validation analysis on the independent dataset. Possible reasons include the small number of original studies, biased negative findings after thresholding in the original studies, different frequency bands, and heterogeneity of PD patients.

## Limitations

A few limitations should be addressed. First, it has been proposed that at least 20 experiments should be included in a meta-analysis [72]. Although the design of RS-fMRI is very similar across studies, too many analytic methods have been applied in studies of brain disorders. Only a small portion of these studies used whole-brain voxel-wise analysis, which is suitable to CB meta-analysis. Reproductive studies using similar analytic methods should be performed. Second, image-based meta-analysis on the unthresholded *t* maps is better than coordinate-based meta-analysis [57]. We suggest that future studies use the same analytic methods, re-analyze the RS-fMRI data from multiple research centers, and perform image-based meta-analysis while taking PD symptoms into account. Third, although the decreased ALFF in the left putamen was the most consistent finding, other brain regions should not be overlooked. In addition to the consistent decreased activity in the left putamen, the current meta-analysis also found increased activity in a few cortical regions, which were consistent with three previous meta-analytic articles [4-6]. It should be noted that these meta-analytic articles, including the current one, recruited the same studies to some extent. Therefore, it is not surprising that these articles found similar abnormal regions. However, most of the abnormal regions were not reproducible in the current validation study on an independent dataset. Future studies could increase sample size and focus on brain regions beyond the putamen.

## Summary

We performed a CB meta-analysis of local activity using RS-fMRI in PD patients and healthy controls and also a validation study using a novel dataset in a frequency-dependent manner. The most consistent result was abnormal ALFF in the left putamen, as evidenced by decreased ALFF in the CB meta-analysis and decreased ALFF of PD in our independent dataset. However, owing to the limited number of original studies that were suitable for CB meta-analysis, our results need to be further validated.

## Potential implications

The consistent finding in the current study is the abnormally decreased ALFF in the left putamen. The precise localization of abnormal brain activity is helpful to identify new targets of focused stimulation, such as deep brain stimulation, transcranial magnetic stimulation, and focused ultrasound stimulation.

## Availability of supporting data

The datasets supporting the results of this article are available in the NeuroImaging Tool & Resources Collaboratory (NITRC) repository, as “PD RS-fMRI meta and validation” [73], and in the *GigaScience* GigaDB repository [74].

## Abbreviations

ALFF: amplitude of low frequency fluctuation; BA: Brodmann area; CB: coordinate based; fALFF: fractional amplitude of low frequency fluctuation; FDR: false discovery rate; fMRI: functional magnetic resonance imaging; FWHM: full-width at half maximum; KCC: Kendall coefficient of concordance; PD: Parkinson's disease; ReHo: regional homogeneity; RS: resting state; SDM: signed differential mapping; SMA: supplementary motor area;

## Ethics, consent, and permissions

The present investigation was performed according to the Declaration of Helsinki and was approved by the Medical Research Ethics Committee at Xuanwu Hospital, Capital Medical University. All participants gave written informed consent prior to participation.

## Competing interests

Y.-F.Z. is an Editorial Board Member of *GigaScience*. All remaining authors have no competing interests.

## Funding

This study was supported by the National Natural Science Foundation of China (81571228 to T.W.; 81271652, 81520108016, and 31471084 to Y.F.Z.), the Ministry of Science and Technology (2016YFC1306503 to T.W.), and the Beijing Municipal Commission of Health and Family Planning (PXM 2017_026283_000002 to T.W.).

## Author contributions

Experimental design: all authors; data collection: J.W., J.R.Z., TW; data analyses: all authors; and writing the article: J.W., Y.F.Z., and T.W.

## Supplementary Material

GIGA-D-18-00065_Original_Submission.pdf

Reviewer_1_Report_(Original_Submission) -- Alejandro De La Vega 03/24/2018 Reviewed

Reviewer_2_Report_(Original_Submission) -- Michael Riedel 04/09/2018 Reviewed
