# Supplementary material for: Consistent decreased activity in the putamen in Parkinson's disease: a meta-analysis and an independent validation of resting-state fMRI
Source: Gigascience. 2018 Jun 18;7(6):giy071. doi: 10.1093/gigascience/giy071 (PMC6025187; doi:10.1093/gigascience/giy071)

GigaScience

Consistent decreased activity in the putamen in Parkinson's disease: A meta-analysis and an independent validation of resting-state fMRI

--Manuscript Draft--

**Manuscript Number:**	GIGA-D-18-00065
**Full Title:**	Consistent decreased activity in the putamen in Parkinson's disease: A meta-analysis and an independent validation of resting-state fMRI
**Article Type:**	Research

Funding Information:

National Natural Science Foundation of China (81571228)	Dr. Tao Wu
National Natural Science Foundation of China (81271652)	Dr. Yu-Feng Zang
National Natural Science Foundation of China (81520108016)	Dr. Yu-Feng Zang
National Natural Science Foundation of China (31471084)	Dr. Yu-Feng Zang
Department of S and T for Social Development (2016YFC1306503)	Dr. Tao Wu
Beijing Municipal Commission of Health and Family Planning (No.PXM 2017_026283_000002)	Dr. Tao Wu
Qian Jiang Distinguished Professor" program	Dr. Yu-Feng Zang

Abstract:

Background: Resting-state functional magnetic resonance imaging (RS-fMRI) has frequently been used to investigate local spontaneous brain activity in Parkinson's disease (PD) in a whole-brain, voxel-wise manner. To quantitatively integrate these studies, we conducted a coordinate-based meta-analysis (CB meta-analysis), using the seed-based d mapping (SDM) method, on 15 studies that used amplitude of low frequency fluctuation (ALFF) and 11 studies that used regional homogeneity (ReHo). All these ALFF and ReHo studies have compared PD patients with healthy controls. We also performed a validation RS-fMRI study of ALFF and ReHo in a frequency-dependent manner for a novel dataset consisting of 49 PD and 49 healthy controls.
Findings: Decreased ALFF was found in the left putamen in PD by meta-analysis. This finding was replicated in our independent validation dataset in the 0.027 - 0.073 Hz band, but not in the conventional frequency band of 0.01 - 0.08 Hz.
Conclusions: Findings from the current study suggested that decreased ALFF in the putamen of PD is the most consistent finding. RS-fMRI is a promising technique for the precise localization of abnormal spontaneous activity in PD. However, more frequency-dependent studies using the same analytical methods are needed to replicate that results.
Trial registration: NCT NCT03439163. Registered 20 February 2018, retrospectively registered.

**Corresponding Author:**	Jue Wang<br>Hangzhou Normal University<br>CHINA
**Corresponding Author Secondary Information:**	
**Corresponding Author's Institution:**	Hangzhou Normal University
**Corresponding Author's Secondary Institution:**	
**First Author:**	Jue Wang

Powered by Editorial Manager® and ProduXion Manager® from Aries Systems Corporation

**First Author Secondary Information:**	
**Order of Authors:**	Jue Wang
	Jia-Rong Zhang
	Yu-Feng Zang
	Tao Wu
**Order of Authors Secondary Information:**	
**Additional Information:**	
**Question**	**Response**
Are you submitting this manuscript to a special series or article collection?	No
**Experimental design and statistics**<br><br>Full details of the experimental design and statistical methods used should be given in the Methods section, as detailed in our Minimum Standards Reporting Checklist. Information essential to interpreting the data presented should be made available in the figure legends.<br><br>Have you included all the information requested in your manuscript?	Yes
**Resources**<br><br>A description of all resources used, including antibodies, cell lines, animals and software tools, with enough information to allow them to be uniquely identified, should be included in the Methods section. Authors are strongly encouraged to cite Research Resource Identifiers (RRIDs) for antibodies, model organisms and tools, where possible.<br><br>Have you included the information requested as detailed in our Minimum Standards Reporting Checklist?	Yes
**Availability of data and materials**<br><br>All datasets and code on which the conclusions of the paper rely must be either included in your submission or deposited in publicly available repositories (where available and ethically appropriate), referencing such data using a unique identifier in the references and in the "Availability of Data and Materials" section of your manuscript.	Yes

Have you have met the above requirement as detailed in our Minimum Standards Reporting Checklist?	

Manuscript Click here to download Manuscript Manuscript(3).docx

Consistent decreased activity in the putamen in Parkinson's disease: A meta-analysis and an independent validation of resting-state fMRI

Running title: Meta-analysis of resting functional imaging in PD

Jue Wang[1,2,3,4], Jia-Rong Zhang[1,5], Yu-Feng Zang[2,3,4]*, and Tao Wu[1,5,6]*

[1]Department of Neurobiology, Key Laboratory on Neurodegenerative Disorders of Ministry of Education, Beijing Institute of Geriatrics, Xuanwu Hospital, Capital Medical University, Beijing, China

[2]Institutes of Psychological Sciences, Hangzhou Normal University, Hangzhou China

[3]Zhejiang Key Laboratory for Research in Assessment of Cognitive Impairments, Hangzhou, China

[4]Center for Cognition and Brain Disorders and the Affiliated Hospital, Hangzhou Normal University, Hangzhou China

[5]Beijing Key Laboratory on Parkinson's Disease, Parkinson Disease Center of Beijing Institute for Brain Disorders, Beijing 10053, China

[6]National Clinical Research Center for Geriatric Disorders, Beijing 100053, China

juefirst@163.com, 771257970@qq.com

*Correspondence: wutao69@163.com; zangyf@gmail.com

ORCID IDs: Jue Wang: 0000-0003-4790-4827; Yu-Feng Zang: 0000-0003-1833-8010

Abstract

Background: Resting-state functional magnetic resonance imaging (RS-fMRI) has frequently been used to investigate local spontaneous brain activity in Parkinson's disease (PD) in a whole-brain, voxel-wise manner. To quantitatively integrate these studies, we conducted a coordinate-based meta-analysis (CB meta-analysis), using the seed-based *d* mapping (SDM) method, on 15 studies that used amplitude of low frequency fluctuation (ALFF) and 11 studies that used regional homogeneity (ReHo). All these ALFF and ReHo studies have compared PD patients with healthy controls. We also performed a validation RS-fMRI study of ALFF and ReHo in a frequency-dependent manner for a novel dataset consisting of 49 PD and 49 healthy controls.

Findings: Decreased ALFF was found in the left putamen in PD by meta-analysis. This finding was replicated in our independent validation dataset in the 0.027 - 0.073 Hz band, but not in the conventional frequency band of 0.01 - 0.08 Hz.

Conclusions: Findings from the current study suggested that decreased ALFF in the putamen of PD is the most consistent finding. RS-fMRI is a promising technique for the precise localization of abnormal spontaneous activity in PD. However, more frequency-dependent studies using the same analytical methods are needed to replicate these results.

Trial registration: NCT NCT03439163. Registered 20 February 2018, retrospectively registered.

Key words: Parkinson's disease; spontaneous brain activity; coordinate-based meta-analysis; putamen

Background

Parkinson's disease (PD) is a progressive neurological degenerative disorder that is characterized by bradykinesia, rigidity, tremor, and postural instability. The main pathological feature of PD is the progressive loss of dopamine neurons in the substantia nigra and putamen [1]. However, it remains unclear how these pathological changes lead to parkinsonian symptoms. To answer this question, many neuroimaging studies using functional magnetic resonance imaging (fMRI) have investigated PD-related neural abnormalities, and found that PD patients showed abnormal activity in the striatum and brainstem, as well as in other brain regions [2]. Most fMRI studies have been focused on motor-related neural changes in PD. Herz et al. conducted a coordinate-based meta-analysis (CB meta-analysis) on motor-task-related functional neuroimaging studies [3], and found a consistently decreased activation in the posterior portion of the putamen in PD patients during motor tasks.

While task-related fMRI holds the advantage of being able to assess specific activation corresponding to specific tasks, different tasks activate different

brain regions and hence the results are less suitable for meta-analysis. RS-fMRI has two intrinsic advantages: noninvasive and task-free. Task-free resting-state is a simpler experimental design for PD investigation. Therefore, RS-fMRI design is very similar across studies, and hence it is more suitable for meta-analysis. There have been three published CB meta-analysis investigations of RS-fMRI studies in PD [4-6]. The CB meta-analysis paper by Tahmasian and colleagues included 28 publications, in which a variety of RS-fMRI analytical methods were used, such as amplitude of low frequency fluctuation (ALFF) or its derivative, fractional ALFF (fALFF); regional homogeneity (ReHo); and various network analytical methods [6]. As a contrast, the analytical methods in the original studies that were included in the two CB meta-analysis papers by Pan and colleagues were very similar, i.e., ReHo [4] and ALFF/fALFF [5], respectively. In between-group comparison studies, ALFF/fALFF and ReHo are usually used in a way of "voxel-wise whole-brain" analysis with very similar preprocessing parameters across studies. Therefore, these studies are suitable for inclusion in meta-analysis [7]. In the CB meta-analysis studies by Pan and colleagues, the authors found decreased ReHo [4] and decreased ALFF [5] in the putamen, which were consistent with the hypothesis of decreased dopaminergic function in the putamen [1].

Frequency-dependent or sub-frequency band analysis is drawing more and more attention in RS-fMRI studies since the work by Zuo and colleagues [8]. They observed a higher RS-fMRI fALFF at a frequency band of 0.027 - 0.073

Hz (namely, slow-4) than that at 0.01 - 0.027 Hz (namely, slow-5) in the basal ganglia which is a critical subcortical area in PD pathology. However, only two of the previous ALFF or ReHo PD studies investigated sub-frequency bands. Hou and colleagues found similar decreased ALFF in the putamen for the two sub-frequency bands [2], but Zhang and colleagues did not find abnormal ALFF in the putamen in any of the two frequency bands [9]. Although higher frequency band (> 0.1 Hz) of RS-fMRI signal could be contaminated by physiological noise including respiratory noise (around 0.3 Hz) and heart beats (around 1.2 Hz), quite a few studies found that RS-fMRI signal at higher frequency bands was of pathophysiological [10-11] and physiological [12] significance. It would be interesting to perform more studies at sub-frequency bands in RS-fMRI studies on PD.

In the current study, we carried out a CB meta-analysis on previous PD resting-state fMRI studies using ALFF/fALFF and ReHo separately, similar to previous meta-analysis [4-5]. However, we also added five papers that were published after the CB meta-analysis papers by Pan and colleagues [4-5]. Further, to validate the results of our CB meta-analysis, we analyzed ALFF and ReHo in the conventional frequency band, as well as in sub-frequency bands, we studied an independent and relatively large cohort of PD patients and healthy controls.

It should be noted that both ALFF and ReHo are metrics for measuring local activity. Both methods have been widely used in studies on brain disorders in a way of “whole-brain voxel-wise” analysis. A study showed that ReHo and ALFF could reveal convergent abnormal local activity in some brain areas [13], but, ReHo and ALFF also detected different brain regions with abnormal brain activity. The two methods are very different mathematically, ReHo depicting the local synchronization of the time-course of neighboring voxels, while ALFF depicting the fluctuation amplitude of every signal time-course. Hence, both separate and combined analyses are necessary. Therefore, in addition to separate meta-analysis on ALFF and ReHo, we were also interested in combining both ALFF and ReHo studies into our meta-analysis. Further, we investigated the frequency-specific features of PD-related brain activity in an independent dataset to improve our understanding of the neurophysiologic al changes underlying PD.

Data Description

The data for the current study included two parts. Part 1 was for a CB meta-analysis including coordinates, *t* value, and sample size. These data were extracted from published papers that used RS-fMRI in PD. Part 2 was for validation purpose, and used RS-fMRI data from 80 patients with PD and 52 healthy participants. After quality control (see details in Analyses section), 49 patients with PD and 49 healthy participants were entered to final analysis. All

the data in the current study can be used for further validation and exploratory studies.

Analyses

Literature search

On June 28, 2017, we conducted a PubMed literature search (www.pubmed.org) using the search strings as follows: ((Parkinson[Title/Abstract]) OR (Parkinson's[Title/Abstract])) AND (("resting-state fMRI" OR ALFF OR ReHo OR "default mode network")). A total of 138 articles were retrieved.

We also reviewed papers and references to retrieve further articles. An additional study, using the Kendall coefficient of concordance (KCC) method, was included in the current meta-analysis after carefully reviewing a recent publication of meta-analysis [4] because KCC is exactly the algorithm of ReHo [14]. Only RS-fMRI studies written in English were eligible. The inclusion criteria were as follows: i) articles reporting original data; ii) analyses using ReHo or ALFF/fALFF and were based on whole brain; iii) articles reporting results on resting-state data in adult PD patients and studies aimed at comparing PD with healthy controls; iv) studies reporting results with coordinates from group comparisons (PD vs. healthy controls) in Montreal Neurological Institute (MNI) or Talairach space; v) patients were in an off-state; and vi) patients were pre-treatment, pre-surgery, and non-comorbid. According to these criteria, two

investigators (Jue Wang, Hong-xiao Wang) independently searched and selected the articles from PubMed. Final decisions were made by a neurologist, Dr. Tao Wu, and 26 RS-fMRI studies (15 studies using ALFF/fALFF [2, 9, 15-27] and 11 studies using ReHo [28-38]) were included in the present CB meta-analysis (Table 1).

Meta-analysis of ALFF and ReHo studies

Seed-based *d* Mapping (formerly Signed Differential Mapping, SDM) software (version 5.141 for Windows) (www.sdmproject.com) was used for meta-analysis. One feature of SDM is “the representation of both positive differences and negative differences in the same map, thus obtaining a signed differential map ('SDM')”. Another feature is “the use of effect sizes (leading to effect-size SDM or 'ES-SDM') (www.sdmproject.com)”. Two directions of abnormality were probed: resting-state activity increases and decreases in PD patients compared with healthy controls. ALFF measures the fluctuation amplitude of the low-frequency band (usually 0.01–0.08 Hz) of a single timecourse [39], and fALFF is the ratio of the ALFF to the fluctuation amplitude of the full-frequency band [40]. ReHo measures the local synchronization of the timecourses of the nearest neighboring voxels (usually 27 voxels) [14]. Although ALFF and ReHo showed significant correlation in most voxels [41], a previous comparison study showed that ALFF and ReHo could detect different abnormal brain areas [13]. Therefore, similar with two previous CB meta-analysis papers [4-5], we first performed meta-analysis separately on

ALFF/fALFF studies (15 studies) and ReHo studies (11 studies, including 13 comparisons) (Table 1) and then combined ALFF and ReHo (see below). The analysis procedure included: i) listing the peak coordinates and *t*-values from each study; ii) using the files prepared in i) to recreate the effect-size maps (standard stereotactic space) of the original studies with 10 Monte Carlo randomizations and full-width at half maximum (FWHM) 20 mm; and iii) generating the mean map in a voxel-wise manner weighted by the sample size, variance, and between-study heterogeneity. A combined threshold of $p < 0.001$ (uncorrected for false discovery rate or FDR) with peak height Z value > 1 was adopted as recommended in the SDM, together with extent threshold > 10 voxels [42-43].

In addition to the above meta-analysis on ALFF and ReHo separately as did by Pan and colleagues [4-5], we further performed a combined meta-analysis of all ALFF and ReHo studies (totally 15 ALFF and 13 ReHo = 28 comparisons. Table 1). Results were also thresholded at $p < 0.001$, uncorrected for FDR, with peak height Z value > 1 and extent threshold > 10 voxels [42-43].

Table 1 (part 1/3. Original papers included in the present meta-analysis study

References	Indices	Sample size (female)	Age (SD)	Disease duration	H&Y (SD)	UPDRS III (SD)	Foci	FWHM	Scanner	Comparison	Medication status
Hou et al., 2014	ALFF	PD 101 (42)	59.84 (7.15)	7.23 (4.42)	1.87 (0.71)	25.54 (11.51)	7	3	3 T	PD OFF vs. HC Slow-4 (0.027–0.073 Hz) #	Off-state
		HC 102 (42)	59.91 (7.09)				4			PD OFF vs. HC Slow-5 (0.01–0.027 Hz) #	
							7			HC vs. PD OFF Slow-4 (0.027–0.073 Hz) #	
							5			HC vs. PD OFF Slow-5 (0.01–0.027 Hz) #	
Kwak et al., 2012	ALFF	PD 24 (2)	64.3 (8)	5.4 (3)	2.2 (0.3)	18.5 (8) *	4	8	3 T	PD OFF vs.HC	Off-state
		HC 24 (5)	63.3 (7)				4			HC vs. PD OFF	
	fALFF						6			PD OFF vs.HC	
							5			HC vs. PD OFF	
Wen et al., 2013	ALFF	PD 16 (8)	60.7 (18.7)	5.6 (7.4)	1.5 (1)	33.8 (24.2)	11	5	3 T	PD-NDep OFF vs.HC	Off-state
		HC 21 (8)	55.4 (16.4)				8			HC vs. PD-NDep OFF	
Yao et al., 2015	ALFF	PD 12 (8)	63.4 (7.4)	8.4 (5.1)	2.8 (0.9)	18.0 (12.9)	5	4	3 T	PDnonVH vs.HC	N/A
		HC 14 (8)	64.1 (4.0)				2			HC vs. PDnonVH	
Zhang et al., 2013	ALFF	PD 82 (47)	59.7 (11.9)	7.05 (6.01)	N/A	20.24 (8.44)	7	8	3 T	PD OFF vs. HC Slow-5 (0.01–0.027 Hz) #	Off-state
		HC 77 (46)	58.6 (8.5)				5			HC vs. PD OFF Slow-4 (0.027–0.073 Hz) #	
							6			HC vs. PD OFF Slow-5 (0.01–0.027 Hz) #	
Luo et al., 2014	ALFF	PD 30 (15)	53.64 (10.18)	2.12 (1.3)	2	26.83 (12.44)	1	8	3 T	PD-NDep OFF vs. HC	Off-state
		HC 30 (15)	51.9 (7.7)								
Chen et al., 2015	ALFF	PD 19 (7)	64.8 (8.34)	6.68 (4.85)	2.13 (0.984)	21.6 (11.6)	5	8	3 T	PIGD vs. HC	N/A
		HC 22 (10)	65.1 (5.0)				5			HC vs. PIGD	
Skidmore et al., 2013	ALFF	PD 14 (3)	62 (9)	N/A	N/A	37 (13)	1	6	3 T	PD vs. HC	Off-state
		HC 15 (6)	65 (13)				7			HC vs. PD	
Hu et al., 2015	fALFF	PD 17 (7)	60.29 (12.03)	3.94 (2.57)	N/A	17.11 (6.12)	3	8	3 T	PD vs. HC	N/A
		HC 20 (9)	58.48 (6.89)								

Table 1 (part 2/3)											
**References**	**Indices**	**Sample size (female)**	**Age (SD)**	**Disease duration**	**H&Y (SD)**	**UPDRS III (SD)**	**Foci**	**FWHM**	**Scanner**	**Comparison**	**Medication status**
Gao et al., 2016	ALFF	PD 16 (6)	64.13 (6.71)	5.69 (4.07)	1.73 (0.57)	16.93 (3.86)	18	8	3 T	HC vs. PD cognitively normal	Off-state
		HC 16 (7)	63.5 (6.49)								
Li et al., 2016	ALFF	PD 16 (10)	62.8 (6.6)	4 (4.3)	2.2 (0.8)	22.1 (12.5)	1	6	3 T	HC vs. PD-nRBD	Off-state
		HC 19 (8)	62.7 (8.1)								
Xiang et al., 2016	ALFF	PD 24 (12)	62.7 (7.4)	7.0 (3.3)	2.2 (0.9)	22.0 (7.0)	3	6	3 T	HC vs. PD OFF	Off-state
		HC 22 (11)	65.6 (6.9)				4			PD OFF vs. HC	
Zhang et al., 2016	ALFF	PD 32 (10)	65 (8.38)	4.04 (3.98)	2.18 (0.67)	21.6 (9.99)	4	6	3 T	PD-NF vs. HC	Off-state
		HC 25 (13)	64.6 (4.49)								
Tang et al., 2017	ALFF/fALFF	PD 51 (24)	53.2 (11)	5.745 (5.026)	2.353 (0.764)	48.59 (23.41)	3	8	3 T	PD vs. HC	Off-state
		HC 50 (29)	51.5 (10.7)								
Possin et al., 2013	fALFF	PD 12 (9)	73.9 (5.9)	9 (7)	N/A	30.8 (14.5)	48	4	3 T	PD vs. HC	Off-state
		HC 12 (11)	72.9 (5.2)								
Choe et al., 2013	ReHo	PD 22 (12)	58.3 (2.4)	3.2 (0.4)	1.6 (0.2)	10.4 ± 1.2	2	9	3 T	PD OFF vs. HC	Off-state
		HC 25 (15)	58.3 (1.7)				1			HC vs. PD OFF	
Wu et al., 2009	ReHo	PD 22 (6)	59.5 (8.1)	4.1 (1.8)	1.7 (0.5)	25.6 (8.1)	11	4	1.5 T	PD OFF vs. HC	Off-state
		HC 22 (6)	59.7 (N/A)				13			HC vs. PD OFF	
Yang et al., 2013	ReHo	PD 17 (7)	60.43 (9.65)	1.6 (1.06)	1.2 (0.33)	20.57 (3.82)	10	4	1.5 T	PD medication-naïve vs. HC	Off-state
		HC 17 (7)	60.73 (8.57)				7			HC vs. PD medication-naïve	
Sheng et al., 2014	ReHo	PD 21 (7)	57.3 (6.1)	4.0 (2.4)	1.95 (0.63)	43.8 (8.2)	3	4	3 T	nD-PD OFF vs. HC	Off-state
		HC 25 (9)	56.7 (5.3)								
Jiang et al., 2015	ReHo	PD 13 (6)	68.46 (6.5)	2.83 (2.38)	2.5 (0.46)	19.31 (8.33)	11	4	3 T	PIGD vs. HC	Off-state
		HC 17 (8)	63.71 (5.21)				16			HC vs. PIGD	
Li et al., 2016	ReHo	PD 23 (12)	63 (7.1)	7 (3.3)	2.2 (0.9)	38 (18.6) *	10	6	3 T	PD vs. HC	Off-state

Table 1 (part 3/3)

References	Indices	Sample size (female)	Age (SD)	Disease duration	H&Y (SD)	UPDRS III (SD)	Foci	FWHM	Scanner	Comparison	Medication status
		HC 20 (9)	65.3 (7.0)				4			HC vs. PD	
Zhang et al., 2015	ReHo	PD 27 (11)	63.38 (9.46)	4.17 (4.07)	2.21 (0.67)	19.88 (6.7)	13	4	3 T	PD-AR vs. HC	Off-state
		HC 26 (15)	59.31 (7.15)				8			HC vs. PD-AR	
Sheng et al., 2016	ReHo	EOPD 18 (8)	45.4 (6.07)	3.04 (1.99)	2.03 (0.78)	16.94 (5.07)	1	4	3 T	Young HC vs. EOPD	Off-state
		Young HC 19 (10)	45.8 (3.55)				1			EPOD vs. Young HC	
		LOPD 21 (9)	63.6 (4.84)	3.1 (1.78)	2.0 (0.62)	18.61 (4.51)	2			Old HC vs. LOPD	Off-state
		Old HC 18 (10)	61.7 (9.73)								
Wen et al., 2016	ReHo	rPD 12 (4)	60.8 (7.02)	5 (N/A)	N/A	28.9 (10.9)	7	4	3 T	rPDpre vs. HC	Off-state
		HC 31 (16)	59.6 (7.65)				2			HC vs. rPDpre	
		lPD 14 (8)	61.4 (7077)	5.5 ((N/A)	N/A	26.4 (15.6)	6			lPDpre vs.HC	Off-state
							8			HC vs. lPDpre	
Yeo et al., 2012	ReHo	PD 12 (6)	53.5 (10.9)	2.67 (2.3)	1.5 (0.6)	7.8 (3.9)	8	9	3 T	HC vs. PD before stimulations	Off-state
		HC 12 (6)	55.9 (9.8)								
Borroni et al., 2015	ReHo	PD 11 (1)	66.3 (3.8)	7.8 (3.1)	N/A	10.7 (5.4)	3	8	1.5 T	HC vs. PD	N/A
		HC 10 (7)	62.2 (8.0)								

Two studies (Hou et al., 2014; Zhang et al., 2013) performed sub-frequency analysis. The two sub-frequency bands were taken into one text file for meta-analysis. *: The score is uncertain whether full UPDRS or part III because we failed to contact the authors.

ALFF: amplitude of low frequency fluctuation; EOPD: early onset PD; fALFF: fractional amplitude of low frequency fluctuation; FWHM: full-width at half maximum; HC: healthy control; H & Y: Hoehn and Yahr scale; lPDpre: pre-left-side-thalamotomy PD; LOPD: late onset PD; nD-PD: non-depressed PD; N-VH PD: no visual hallucinations PD; PD: Pakinson's disease; PD-AR: akinetic-rigid PD; PD-nRBD: no rapid eye movement sleep behavior disorder PD; PD-NDep: non-depressed PD; PD OFF: PD off medication; PIGD: postural instability gait difficulty PD; ReHo: regional homogeneity; rPDpre: pre-right-side-thalamotomy PD; UPDRS: unified Parkinson's disease rating scale.

Validation study of ALFF and ReHo on an independent dataset of RS-fMRI

Participants

This validation study contains RS-fMRI data of 80 patients with PD and 52 healthy participants. After head motion control, 12 patients were excluded, and matching for age and gender was conducted. A total of 98 right-handed participants, made up of 49 PD patients (26 females) and 49 age- and gender-matched healthy controls (26 females) were enrolled in the final analysis (mean age ± SD: 62.3 y ± 8.0, 61.8 y ± 8.3, respectively; Table 2). The PD diagnoses were based on the UK Parkinson's Disease Society Brain Bank Clinical Diagnostic Criteria [44]. Patients were assessed using the UPDRS III [45] and Hoehn and Yahr disability scale [46]. Exclusion criteria included history of head trauma, substance abuse, or psychiatric disorder. For healthy controls, additional exclusion criteria included any history of neuropsychiatric disorders. The present investigation was performed according to the Declaration of Helsinki and was approved by the Medical Research Ethics Committee at Xuanwu Hospital, Capital Medical University. All participants gave written informed consent prior to participation.

Table 2. Demographic characteristics.

	**PD**	**Control**
Male/Femal	N=49 (23/26)	N=49 (23/26)
Age (Y)	62.3 ± 8.0	61.8 ± 8.3
Disease duration (Y)	5.5 ± 3.8	-
Disease stage (H&Y)	1.9 ± 0.7	-
UPDRS III	23.3 ± 11.0	-
MMSE	28.3 ± 1.6	-

H & Y: Hoehn and Yahr scale; MMSE: Mini-mental State Examination; PD: Pakinson's disease; UPDRS: unified Parkinson's disease rating scale.

Data acquisition

fMRI data were acquired on a 3T MR scanner (Trio system; Siemens Magnetom scanner, Erlangen, Germany) with gradient-echo echo-plannar imaging sequences. Whole brain fMRI scanning with three slightly different parameters was carried out (see Table 3). All participants were instructed to keep their eyes closed, relax, remain motionless, not think of anything in particular, and not fall asleep. Foam pads were used to minimize head motion.

Table 3. Parameters of the three resting-state fMRI datasets.

	**Dataset 1.**	**Dataset 2.**	**Dataset 3.**
TR (ms)	2000	2000	2000
TE (ms)	30	40	40
FOV	220 mm× 220 mm	256 mm× 256 mm	256 mm× 256 mm
matrix	64 × 64	64 × 64	64 × 64
Flip angle	90	90	90
Slice thickness	3	4	4
gap	0.5	1	1
slices	32	28	28
volumes	180	239	300

FOV: field of view; TR: time of repetition; TE: time of echo.

Data analyses

The minimum time-points were 180 (Dataset 1, Table 3). The extra time-points of dataset 2 and 3 were discarded and hence 180 time-points were left. The RS-fMRI data preprocessing included the following steps: i) discarding the first 10 volumes to allow the signal to reach equilibrium and the subjects to adapt to the circumstances; ii) correcting for the acquisition time delay between slices; iii) rigid-body realignment for estimation and correction of the motion displacement (participants whose head motion exceeded 2 mm

in translation or 2 degrees in rotation in any direction were excluded); iv) normalization to MNI space using the echo-plannar imaging (EPI) template in Statistical Parametric Mapping (SPM) 8 (http://www.fil.ion.ucl.ac.uk/spm/); v) regressing out of the six motion parameters; vi) removal of the linear trend; and vii) band-pass filtering for five frequency bands (0–0.01, 0.01–0.027, 0.027–0.073, 0.073–0.198, and 0.198–0.25 Hz, as well as 0.01–0.08 Hz). Most of the previous PD studies investigating amplitude of low frequency fluctuation used ALFF but not fALFF. We therefore analyzed ALFF only. For calculating ALFF, data were further smoothed, with a Gaussian kernel of 6 mm FWHM, and ALFF was then calculated using the REST toolkit (http://www.restfmri.net). For ReHo, the calculation was performed first, and then the 6 mm FWHM smoothing was carried out on the ReHo maps.

Statistical analyses

Two-sample *t*-tests were performed to explore the differences between the two groups in a voxel-wise manner for ALFF and ReHo, respectively, for each sub-frequency band as well for the conventional frequency band (0.01 – 0.08 Hz). Monte Carlo simulation (AlphaSim) was applied for the multiple comparison correction within a whole brain mask by using DPABI_V3.0 (http://www.rfmri.org/dpabi) (DPABI, RRID:SCR_010501) software [47]. DPABI estimates the smoothness of each statistic map and hence yields effective kernel size of smoothness for each map. And then the smoothness was used for the correction. It is believed that simply taking the size of Gaussian kernel

that was applied during preprocessing to AlphaSim is incorrect [48]. DPABI prevents that short of error by estimated effective smoothness. Although Monte Carlo simulations in DPABI are based on AFNI's 3dClustSim, the specific algorithm used is not by the bug reported in Eklund *et al.*, 2016 since version 1.2_141101[49-50]. The corresponding estimated smoothness and minimal cluster size was listed in Table 4 for each frequency band of ALFF and ReHo. The voxel-level *p* value was set at < 0.001 as recommended [49]. The corrected *p* value was < 0.05.

Table 4. The estimated smoothness and cluster size of T maps (PD vs. healthy controls based on validation study)

Frequency Band	Estimated Smoothness (mm)			Cluster Size (number of voxels)
	FWHM x	FWHM y	FWHM z	
**ALFF**				
0.01-0.08 Hz	10.42	11.20	10.68	46
0-0.01 Hz	8.81	9.23	9.03	30
0.01-0.027 Hz	8.80	9.15	9.04	31
0.027-0.073 Hz	9.53	9.86	9.79	35
0.073-0.198 Hz	7.87	8.22	7.82	23
0.198-0.25 Hz	7.41	7.61	7.04	19
**ReHo**				
0.01-0.08 Hz	12.24	13.03	13.37	64
0-0.01 Hz	13.07	14.35	13.80	74
0.01-0.027 Hz	12.34	12.55	12.68	62
0.027-0.073 Hz	12.39	13.12	13.49	69
0.073-0.198 Hz	12.07	13.54	12.93	65
0.198-0.25 Hz	11.53	12.18	11.84	56

Findings

Meta-analysis on ALFF and ReHo studies

In the meta-analysis of SDM for ALFF, an increased ALFF in PD patients compared with controls was found in the right inferior temporal gyrus. A

decreased ALFF in PD patients compared with controls was found in the left pallidum/putamen and the right cuneus cortex (Fig. 1, Table 5).

Meanwhile, using SDM for ReHo, increased ReHo was observed in the bilateral IPL and the right superior frontal gyrus/pre-SMA (BA 9) in PD patients. In addition, decreased ReHo was observed in the right putamen and right precentral gyrus (BA 6) in PD patients (Fig. 2, Table 5).

In the meta-analysis of SDM for combination of ALFF and ReHo, increased spontaneous brain activities in PD patients compared with controls were found in the right inferior temporal gyrus and right brainstem. While decreased spontaneous brain activities were found in the left pallidum/putamen, and the right insula (BA 47) (Fig. 3, Table 5).

Table 5. The brain regions showing differences between PD and healthy controls based on meta-analysis

Brain Region	BA	MNI (X Y Z)			SDM-Z value	Cluster Size (mm^3)	P value
**SDM (15 ALFF/fALFF studies)**							
**PD > Controls**							
Right Inferior Longitudinal Fascicules		42	-28	-14	3.37	6256	0.000000894
**PD < Controls**							
Left Pallidum/Putamen		-22	4	6	3.08	2160	0.000015318
Right Cuneus Cortex	19	6	-88	26	2.39	80	0.000558496
**SDM (11 ReHo studies)**							
**PD > Controls**							
Left Inferior Parietal Lobule	39	-44	-66	38	2.55	4552	0.000009179
Right superior frontal gyrus/pre-SMA	9	10	38	48	2.24	2120	0.0000844
Right Inferior Parietal Lobule	40	56	-42	40	1.95	360	0.000498116
**PD < Controls**							
Right Putamen/Insula	48	36	-4	4	2.46	8744	0.000002623
Right Precentral Gyrus	6	44	-4	48	1.78	312	0.000464916
**SDM (15 ALFF/fALFF studies+11 ReHo studies)**							
**PD > Controls**							
Right Inferior Longitudinal Fascicules		44	-30	-16	3.04	2168	0.000040650
Brainstem		2	-28	-26	2.58	224	0.000389218
**PD < Controls**							
Left Pallidum/Putamen		-22	10	12	2.92	2504	0.000040233
Right Insula	47	30	24	-2	2.51	248	0.000307441

ALFF: amplitude of low frequency fluctuation; BA: Brodmann area; fALFF: fractional amplitude of low frequency fluctuation; MNI: Montreal Neurological Institute; PD: Parkinson's disease; ReHo: regional homogeneity; SDM: seed-based *d* mapping; SMA: supplementary motor area.

Results of the validation study of ALFF and ReHo

Compared with healthy controls, PD patients had decreased ALFF in the bilateral putamen and right fusiform at 0.027–0.073 Hz (Fig. 4, Table 6). PD patients also had decreased ReHo in the left inferior occipital gyrus at 0–0.01 Hz, and increased ReHo in the right middle frontal gyrus at 0.073-0.198 Hz and 0.198–0.25 Hz (Fig. 4, Table 6). The other frequency bands, including

conventional 0.01 – 0.08 Hz, did not show significant difference of ALFF or ReHo. Among these brain regions, the decreased ALFF at 0.027–0.073 Hz in the left putamen was overlapped with our findings of decreased ALFF in the meta-analysis.

Table 6. The brain regions of validation study showing differences between PD and healthy controls

Brain Region	Frequency Band (Hz)	BA	MNI (X Y Z)			Peak t value	Cluster Size (mm^3)	P value
**ALFF**								
**PD < Controls**								
Right Putamen	0.027 – 0.073		24	12	6	4.22	1296	<0.001
Left Putamen	0.027 – 0.073		-21	9	3	4.90	1188	<0.001
Right Fusiform	0.027 – 0.073	19	30	-75	-15	4.53	1512	<0.001
White Matter			21	-9	27	4.79	1512	<0.001
**ReHo**								
**PD < Controls**								
Left Inferior Occipital Gyrus	0 – 0.01	19	-33	-87	-12	4.37	2322	<0.001
**PD > Controls**								
Right Middle Frontal Gyrus	0.073 – 0.198	46	30	21	39	4.97	2187	<0.001
Right Middle Frontal Gyrus	0.198 – 0.25	9	33	27	48	4.83	1782	<0.001

ALFF: amplitude of low frequency fluctuation; MNI: Montreal Neurological Institute; ReHo: regional homogeneity.

Discussion

Using the SDM meta-analysis, we detected some characteristic PD-related neural changes in the resting state; for example, there was altered local activity in the putamen and the pre-SMA. A finding of decreased ALFF in the left putamen from our validation study was very consistent with the results of our CB meta-analysis, which was also consistent with findings from a previous CB meta-analysis [5]. The increased ReHo in the validation dataset was not consistent with our CB meta-analysis results and the previous one [4], however.

Methodology: whole-brain voxel-wise comparison and image-based meta-analysis

As compared with positron emission tomography, RS-fMRI has the advantages of lower cost, better temporal resolution, and no ionizing radiation. Since 2009 when the first PD RS-fMRI paper was published [29], there have been approximately 150 research articles published using RS-fMRI in PD. To ensure that the analytical methods were as similar as possible, two previous CB meta-analysis papers included only ALFF [5] or ReHo [4], because many other analytical methods are not “whole-brain voxel-wise” and hence not suitable to CB meta-analysis. For example, the “seed” location of seed-based functional connectivity varies greatly across studies, and for independent component analysis, researchers may be interested in any network or component. Likewise, a very small portion of the existing graph theory studies are “whole-brain voxel-wise” [51]; instead, most of them are region-based due to the computational cost of this method. Unfortunately, because only a small portion of RS-fMRI studies have used "whole-brain voxel-wise" analytic methods, the two previous CB meta-analysis studies have included only a limited number (10 or less) of papers in which the same analytical methods were used. After our careful screening of PD RS-fMRI papers, we found a few additional eligible studies which used "whole-brain voxel-wise" analytic methods; i.e., ALFF/fALFF (15 papers) and ReHo (11 papers; Table 1). It has been suggested that, for meta-analysis, unthresholded effect size maps

(named image-based) was better than coordinate-based meta-analysis [52]. Therefore, effect size maps from “whole-brain voxel-wise” comparison should be widely performed in future RS-fMRI studies.

Methodology: ALFF vs. ReHo

ALFF measures the amplitude of fluctuation of every single voxel, while ReHo measures the local synchronization of nearest neighboring voxels. The two metrics are the two most widely used methods for depicting local activity [53]. And it has been shown that the ALFF and ReHo were among the RS-fMRI metrics which have the highest test-retest reliability [54]. Although the ALFF and ReHo methods are mathematically different, both methods measure the local activity of spontaneous brain activity. A previous study compared the two methods in attention deficit hyperactivity disorder [13] and found a few convergent abnormal regions for ALFF and ReHo, albeit some divergent abnormal activity existed. Therefore, we performed a combined meta-analysis on all ALFF and ReHo studies. It was shown that, the results of combined meta-analysis of all ALFF and ReHo studies looked like complimentary results of separate meta-analysis mutually between ALFF and ReHo. An interesting result is the decreased spontaneous activity in the bilateral putamen. And these results were replicated in the independent validation dataset. Comparing the results of ALFF and ReHo either by meta-analysis or by independent validation analysis, almost no convergent results were found for the two metrics. More studies are needed in the future

to compare the two methods.

Consistent decreased ALFF in the left putamen

There was decreased ALFF in the left putamen in PD patients in both our validation dataset and CB meta-analysis investigation. These findings align with previous finding that the striatal dysfunction in PD has been consistently reported in previous studies [55-62]. And it has been established that dopamine uptake is reduced in the putamen in PD [63], which is a critical factor that leads to major parkinsonian symptoms. Cells loss from the substantia nigra in PD results in dopamine expending in the striatum, with putamen being affected [64-65]. ^{18}F-dopa uptake in putamen in PD has been reported to associate with the clinical severity of locomotor disability, and ^{18}F-dopa reductions in putamen associate well with the degree of rigidity and bradykinesia [63, 66]. A recent meta-analysis of motor-related task fMRI studies also found decreased activity in the putamen in PD patients [3]. Notably, in our validation dataset, we found that the decreased ALFF was mainly found in a sub-frequency band of 0.027–0.073 Hz, namely slow-4 [8]. Either ALFF or ReHo abnormality in the left putamen was not found in the conventional frequency band of 0.01 – 0.08 Hz. We suggest that future RS-fMRI studies should pay more attention on sub-frequency analysis to validate this finding. Further, the physiological importance of each sub-frequency band of RS-fMRI should also be more investigated.

Inconsistent ReHo and ALFF findings

There was decreased ReHo in the right putamen in PD patients in both our CB meta-analysis and a previous CB meta-analysis [4]. But no ReHo changes were found in the validation dataset (Fig. 4). This discrepancy in findings between ALFF and ReHo might be explained by the differences in the two methods as discussion in the section of “*Methodology: ALFF vs. ReHo*”. Further, except for the consistent results in the left putamen in ALFF, most meta-analysis results were not reproducible in the validation analysis on the independent dataset. Possible reasons include, at least, small number of original studies, biased negative findings after thresholding in the original studies, different frequency bands, as well as heterogeneity of PD patients.

Limitations

A few limitations should be addressed. Firstly, it has been proposed that at least 20 experiments should be included in a meta-analysis [67]. Although the design of RS-fMRI is very similar across studies, too many analytic methods have been applied in studies of brain disorders. Only a small part of these studies has utilized “whole-brain voxel-wise” analysis which is suitable to CB meta-analysis. Reproductive studies using similar analytic methods should be widely carried out. Secondly, the image-based meta-analysis on the unthresholded *t* maps is better than coordinate-based meta-analysis [52]. We thus suggest that future studies to use the same analytic methods, re-analyze the RS-fMRI data from multiple research centers, and perform image-based meta-analysis, while taking PD symptoms into account. Thirdly,

although the decreased ALFF in the left putamen was the most consistent finding, other brain regions should not be overlooked. Actually, in addition to the consistent decreased activity in the left putamen, the current meta-analysis also found increased activity in a few cortical regions, which were consistent with the 3 previous meta-analytic papers [4-6]. It should be noted that these meta-analytic papers, including the current one, recruited same studies in some extent. Therefore, it is not surprising that these papers found similar abnormal regions. But most of the abnormal regions were not reproducible in the current validation study on an independent dataset. Future studies could increase sample size and focus on brain regions beyond the putamen.

Summary

We performed a CB meta-analysis of local activity using RS-fMRI in PD and healthy controls, and also a validation study using a novel dataset in a frequency-dependent manner. The most consistent result was abnormal ALFF in the left putamen, as evidenced by decreased ALFF in the CB meta-analysis and decreased ALFF of PD in our independent dataset. However, owing to the limited number of original studies that were suitable for CB meta-analysis, our results need to be further validated.

Potential implications

The consistent finding in the current study is the abnormally decreased ALFF in the left putamen. The precise localization of abnormal brain activity is

helpful to identify new targets of focused stimulation, such as deep brain stimulation, transcranial magnetic stimulation, and focused ultrasound stimulation.

Availability of supporting data and materials

The datasets supporting the results of this article are available in the NITRC repository, named as “PD RS-fMRI meta and validation” [68], and in the *GigaScience* GigaDB repository[69].

Acknowledgments

We thank Bronwen Gardner, PhD, from Liwen Bianji, Edanz Editing China (www.liwenbianji.cn/ac), for editing the English text of a draft of this manuscript. We thank Miss Hong-Xiao Wang, from Hangzhou Normal University China, for screening the literatures from PubMed. We also thank an anonymous reviewer for his/her constructive suggestion to perform a combined meta-analysis on all ALFF and ReHo studies.

Declarations

List of abbreviations

ALFF: amplitude of low frequency fluctuation

CB meta-analysis: coordinate-based meta-analysis

EPI: echo-plannar imaging

fALFF: fractional amplitude of low frequency fluctuation

FDR: false discovery rate

fMRI: functional magnetic resonance imaging

FWHM: full-width at half maximum

IPL: inferior parietal lobule

KCC: Kendall coefficient of concordance

MNI: Montreal Neurological Institute

PD: Parkinson’s disease

ReHo: regional homogeneity

ROI: region of interest

RS-fMRI: resting-state functional magnetic resonance imaging

SDM: seed-based *d* mapping

SMA: supplementary motor area

SPM: Statistical Parametric Mapping

UPDRS: unified Parkinson's disease rating scale

Ethics, consent and permissions

The present investigation was performed according to the Declaration of Helsinki and was approved by the Medical Research Ethics Committee at Xuanwu Hospital, Capital Medical University. All participants gave written informed consent prior to participation.

Competing interests

The author Dr. Yu-Feng Zang is an editorial member of GigaScience. All

authors declare no other conflicts of interest.

Funding

This study was supported by the National Natural Science Foundation of China (81571228 to TW; 81271652, 81520108016, and 31471084 to YFZ); the Ministry of Science and Technology (2016YFC1306503) to TW, and Beijing Municipal Commission of Health and Family Planning, No.PXM 2017_026283_000002 to TW. Dr. Zang is partly supported by the “Qian Jiang Distinguished Professor” program.

Authors' contributions

Experimental design: all authors; Data collection: JW, JRZ, TW; Data analyses: all authors; Paper writing: JW, YFZ, and TW.

Figure legends

Fig. 1 ALFF/fALFF differences between PD and healthy controls in CB meta-analysis (*p* < *0.001*, uncorrected for FDR, peak height Z value > 1, extent threshold > 10 voxels). The warm color represents an increased SDM value and the cold color represents a decreased SDM value in PD. ALFF: amplitude of low frequency fluctuation; CB meta-analysis: coordinate-based meta-analysis; fALFF: fractional amplitude of low frequency fluctuation; FDR: false discovery rate; PD: Parkinson’s disease; SDM: seed-based d mapping.

Fig. 2 ReHo differences between PD and healthy controls in CB meta-analysis ($p < 0.001$, uncorrected for FDR, peak height Z value > 1, extent threshold > 10 voxels). The warm color represents an increased SDM value and the cold color represents a decreased SDM value in PD. ReHo: regional homogeneity; CB meta-analysis: coordinate-based meta-analysis; FDR: false discovery rate; PD: Parkinson's disease; SDM: seed-based d mapping.

Fig. 3 Combined ALFF and ReHo differences between PD and healthy controls in CB meta-analysis ($p < 0.001$, uncorrected for FDR, peak height Z value > 1, extent threshold > 10 voxels). The warm color represents an increased SDM value and the cold color represents a decreased SDM value in PD. ALFF: amplitude of low frequency fluctuation; CB meta-analysis: coordinate-based meta-analysis; fALFF: fractional amplitude of low frequency fluctuation; ReHo: regional homogeneity; FDR: false discovery rate; PD: Parkinson's disease; SDM: seed-based d mapping.

Fig. 4 ALFF/ReHo differences between PD and healthy controls in the validation study. The warm color represents an increased ALFF/ReHo and the cold color represents a decreased ALFF/ReHo in PD (voxel-level $p < 0.001$, corrected $p < 0.05$). ALFF: amplitude of low frequency fluctuation; ReHo: regional homogeneity; PD: Parkinson's disease.

References

1. Politis M. Neuroimaging in Parkinson disease: from research setting to clinical practice. Nat Rev Neurol. 2014;10:708-22.
2. Hou Y, Wu X, Hallett M, Chan P, Wu T. Frequency-dependent neural activity in Parkinson's disease. Human brain mapping. 2014;35:5815-33.
3. Herz DM, Eickhoff SB, Lokkegaard A, Siebner HR. Functional neuroimaging of motor control in Parkinson's disease: a meta-analysis. Human brain mapping. 2014;35:3227-37.
4. Pan P, Zhan H, Xia M, Zhang Y, Guan D, Xu Y. Aberrant regional homogeneity in Parkinson's disease: A voxel-wise meta-analysis of resting-state functional magnetic resonance imaging studies. Neurosci Biobehav Rev. 2017;72:223-31.
5. Pan P, Zhang Y, Liu Y, Zhang H, Guan D, Xu Y. Abnormalities of regional brain function in Parkinson's disease: a meta-analysis of resting state functional magnetic resonance imaging studies. Sci Rep. 2017;7:40469.
6. Tahmasian M, Eickhoff SB, Giehl K, Schwartz F, Herz DM, Drzezga A, et al. Resting-state functional reorganization in Parkinson's disease: An activation likelihood estimation meta-analysis. Cortex. 2017;92:119-38.
7. Zang YF, Zuo XN, Milham M, Hallett M. Toward a Meta-Analytic Synthesis of the Resting-State fMRI Literature for Clinical Populations. Biomed Res Int. 2015;2015:435265.
8. Zuo XN, Di Martino A, Kelly C, Shehzad ZE, Gee DG, Klein DF, et al. The oscillating brain: complex and reliable. Neuroimage. 2010;49:1432-45.
9. Zhang J, Wei L, Hu X, Zhang Y, Zhou D, Li C, et al. Specific frequency band of amplitude low-frequency fl uctuation predicts Parkinson's disease. Behavioural brain research. 2013;252:18-23.
10. Malinen S, Vartiainen N, Hlushchuk Y, Koskinen M, Ramkumar P, Forss N, et al. Aberrant temporal and spatial brain activity during rest in patients with chronic pain. Proc Natl Acad Sci U S A. 2010;107:6493-7.
11. Otti A, Guendel H, Wohlschlager A, Zimmer C, Noll-Hussong M. Frequency shifts in the anterior default mode network and the salience network in chronic pain disorder. BMC psychiatry. 2013;13:84.
12. Yuan BK, Wang J, Zang YF, Liu DQ. Amplitude differences in high-frequency fMRI signals between eyes open and eyes closed resting states. Front Hum Neurosci. 2014;8:503.
13. An L, Cao QJ, Sui MQ, Sun L, Zou QH, Zang YF, et al. Local synchronization and amplitude of the fluctuation of spontaneous brain activity in attention-deficit/hyperactivity disorder: a resting-state fMRI study. Neurosci Bull. 2013;29:603-13.
14. Zang Y, Jiang T, Lu Y, He Y, Tian L. Regional homogeneity approach to fMRI data analysis. Neuroimage. 2004;22:394-400.
15. Kwak Y, Peltier SJ, Bohnen NI, Muller ML, Dayalu P, Seidler RD. L-DOPA changes spontaneous low-frequency BOLD signal oscillations in Parkinson's disease: a resting state fMRI study. Frontiers in systems neuroscience. 2012;6:52.
16. Wen X, Wu X, Liu J, Li K, Yao L. Abnormal baseline brain activity in non-depressed Parkinson's disease and depressed Parkinson's disease: a resting-state functional magnetic resonance imaging study. PloS one. 2013;8:e63691.

17. Yao N, Pang S, Cheung C, Chang RS, Lau KK, Suckling J, et al. Resting activity in visual and corticostriatal pathways in Parkinson's disease with hallucinations. Parkinsonism & related disorders. 2015;21:131-7.

18. Luo C, Chen Q, Song W, Chen K, Guo X, Yang J, et al. Resting-state fMRI study on drug-naive patients with Parkinson's disease and with depression. Journal of neurology, neurosurgery, and psychiatry. 2014;85:675-83.

19. Chen HM, Wang ZJ, Fang JP, Gao LY, Ma LY, Wu T, et al. Different patterns of spontaneous brain activity between tremor-dominant and postural instability/gait difficulty subtypes of Parkinson's disease: a resting-state fMRI study. CNS Neurosci Ther. 2015;21:855-66.

20. Skidmore FM, Yang M, Baxter L, Von Deneen KM, Collingwood J, He G, et al. Reliability analysis of the resting state can sensitively and specifically identify the presence of Parkinson disease. Neuroimage. 2013;75:249-61.

21. Hu XF, Zhang JQ, Jiang XM, Zhou CY, Wei LQ, Yin XT, et al. Amplitude of low-frequency oscillations in Parkinson's disease: a 2-year longitudinal resting-state functional magnetic resonance imaging study. Chinese medical journal. 2015;128:593-601.

22. Gao LL, Wu XM, Zhang JR, Chan P, Wu T. Brain activity in Parkinson's disease patients with mild cognitive impairment. Sci Bull. 2016;61:1876-83.

23. Li D, Huang P, Zang Y, Lou Y, Cen Z, Gu Q, et al. Abnormal baseline brain activity in Parkinson's disease with and without REM sleep behavior disorder: A resting-state functional MRI study. J Magn Reson Imaging. 2016DOI 10.1002/jmri.25571.

24. Xiang J, Jia X, Li H, Qin J, Liang P, Li K. Altered Spontaneous Brain Activity in Cortical and Subcortical Regions in Parkinson's Disease. Parkinsons Dis. 2016;2016:5246021.

25. Zhang JJ, Ding J, Li JY, Wang M, Yuan YS, Zhang L, et al. Abnormal Resting-State Neural Activity and Connectivity of Fatigue in Parkinson's Disease. CNS Neurosci Ther. 2017;23:241-47.

26. Tang Y, Meng L, Wan CM, Liu ZH, Liao WH, Yan XX, et al. Identifying the presence of Parkinson's disease using low-frequency fluctuations in BOLD signals. Neuroscience letters. 2017;645:1-6.

27. Possin KL, Kang GA, Guo C, Fine EM, Trujillo AJ, Racine CA, et al. Rivastigmine is associated with restoration of left frontal brain activity in Parkinson's disease. Movement disorders : official journal of the Movement Disorder Society. 2013;28:1384-90.

28. Choe IH, Yeo S, Chung KC, Kim SH, Lim S. Decreased and increased cerebral regional homogeneity in early Parkinson's disease. Brain research. 2013;1527:230-7.

29. Wu T, Long X, Zang Y, Wang L, Hallett M, Li K, et al. Regional homogeneity changes in patients with Parkinson's disease. Human brain mapping. 2009;30:1502-10.

30. Yang H, Zhou XJ, Zhang MM, Zheng XN, Zhao YL, Wang J. Changes in spontaneous brain activity in early Parkinson's disease. Neuroscience letters. 2013;549:24-8.

31. Sheng K, Fang W, Su M, Li R, Zou D, Han Y, et al. Altered spontaneous brain activity in patients with Parkinson's disease accompanied by depressive symptoms, as revealed by regional homogeneity and functional connectivity in the prefrontal-limbic system. PloS one. 2014;9:e84705.

32. Jiang S, Wang M, Zhang L, Yuan Y, Tong Q, Ding J, et al. Regional homogeneity alterations differentiate between tremor dominant and postural instability gait difficulty subtypes of Parkinson's disease. J Neural Transm (Vienna). 2016;123:219-29.

33. Li Y, Liang P, Jia X, Li K. Abnormal regional homogeneity in Parkinson's disease: a resting state fMRI study. Clin Radiol. 2016;71:e28-34.
34. Zhang J, Wei L, Hu X, Xie B, Zhang Y, Wu GR, et al. Akinetic-rigid and tremor-dominant Parkinson's disease patients show different patterns of intrinsic brain activity. Parkinsonism & related disorders. 2015;21:23-30.
35. Sheng K, Fang W, Zhu Y, Shuai G, Zou D, Su M, et al. Different Alterations of Cerebral Regional Homogeneity in Early-Onset and Late-Onset Parkinson's Disease. Front Aging Neurosci. 2016;8:165.
36. Wen Z, Zhang J, Li J, Dai J, Lin F, Wu G. Altered Activation in Cerebellum Contralateral to Unilateral Thalamotomy May Mediate Tremor Suppression in Parkinson's Disease: A Short-Term Regional Homogeneity fMRI Study. PloS one. 2016;11:e0157562.
37. Yeo S, Lim S, Choe IH, Choi YG, Chung KC, Jahng GH, et al. Acupuncture stimulation on GB34 activates neural responses associated with Parkinson's disease. CNS Neurosci Ther. 2012;18:781-90.
38. Borroni B, Premi E, Formenti A, Turrone R, Alberici A, Cottini E, et al. Structural and functional imaging study in dementia with Lewy bodies and Parkinson's disease dementia. Parkinsonism & related disorders. 2015;21:1049-55.
39. Zang YF, He Y, Zhu CZ, Cao QJ, Sui MQ, Liang M, et al. Altered baseline brain activity in children with ADHD revealed by resting-state functional MRI. Brain Dev. 2007;29:83-91.
40. Zou QH, Zhu CZ, Yang Y, Zuo XN, Long XY, Cao QJ, et al. An improved approach to detection of amplitude of low-frequency fluctuation (ALFF) for resting-state fMRI: fractional ALFF. J Neurosci Methods. 2008;172:137-41.
41. Aiello M, Salvatore E, Cachia A, Pappata S, Cavaliere C, Prinster A, et al. Relationship between simultaneously acquired resting-state regional cerebral glucose metabolism and functional MRI: a PET/MR hybrid scanner study. Neuroimage. 2015;113:111-21.
42. Lim L, Radua J, Rubia K. Gray matter abnormalities in childhood maltreatment: a voxel-wise meta-analysis. Am J Psychiatry. 2014;171:854-63.
43. Radua J, Mataix-Cols D, Phillips ML, El-Hage W, Kronhaus DM, Cardoner N, et al. A new meta-analytic method for neuroimaging studies that combines reported peak coordinates and statistical parametric maps. European psychiatry : the journal of the Association of European Psychiatrists. 2012;27:605-11.
44. Hughes AJ, Daniel SE, Kilford L, Lees AJ. Accuracy of clinical diagnosis of idiopathic Parkinson's disease: a clinico-pathological study of 100 cases. Journal of neurology, neurosurgery, and psychiatry. 1992;55:181-4.
45. Lang AE, Fahn S. Assessment of Parkinson's disease. In: Munsat TL, editor. Quantification of Neurological Deficit. 1989DOI:285-309.
46. Hoehn MM, Yahr MD. Parkinsonism: onset, progression and mortality. Neurology. 1967;17:427-42.
47. Yan CG, Wang XD, Zuo XN, Zang YF. DPABI: Data Processing & Analysis for (Resting-State) Brain Imaging. Neuroinformatics. 2016;14:339-51.
48. Bennett CM, Wolford GL, Miller MB. The principled control of false positives in neuroimaging. Social cognitive and affective neuroscience. 2009;4:417-22.
49. Eklund A, Nichols TE, Knutsson H. Cluster failure: Why fMRI inferences for spatial extent have inflated false-positive rates. Proc Natl Acad Sci U S A. 2016;113:7900-5.

50. Chen X, Lu B, Yan CG. Reproducibility of R-fMRI metrics on the impact of different strategies for multiple comparison correction and sample sizes. Human brain mapping. 2018;39:300-18.
51. Lou Y, Huang P, Li D, Cen Z, Wang B, Gao J, et al. Altered brain network centrality in depressed Parkinson's disease patients. Movement disorders : official journal of the Movement Disorder Society. 2015;30:1777-84.
52. Salimi-Khorshidi G, Smith SM, Keltner JR, Wager TD, Nichols TE. Meta-analysis of neuroimaging data: a comparison of image-based and coordinate-based pooling of studies. Neuroimage. 2009;45:810-23.
53. Di Martino A, Fair DA, Kelly C, Satterthwaite TD, Castellanos FX, Thomason ME, et al. Unraveling the miswired connectome: a developmental perspective. Neuron. 2014;83:1335-53.
54. Zuo XN, Xing XX. Test-retest reliabilities of resting-state FMRI measurements in human brain functional connectomics: a systems neuroscience perspective. Neurosci Biobehav Rev. 2014;45:100-18.
55. Wu T, Liu J, Zhang H, Hallett M, Zheng Z, Chan P. Attention to Automatic Movements in Parkinson's Disease: Modified Automatic Mode in the Striatum. Cereb Cortex. 2015;25:3330-42.
56. Wu T, Zhang J, Hallett M, Feng T, Hou Y, Chan P. Neural correlates underlying micrographia in Parkinson's disease. Brain : a journal of neurology. 2016;139:144-60.
57. Wu T, Wang L, Chen Y, Zhao C, Li K, Chan P. Changes of functional connectivity of the motor network in the resting state in Parkinson's disease. Neuroscience letters. 2009;460:6-10.
58. Wu T, Wang L, Hallett M, Chen Y, Li K, Chan P. Effective connectivity of brain networks during self-initiated movement in Parkinson's disease. Neuroimage. 2011;55:204-15.
59. Yu H, Sternad D, Corcos DM, Vaillancourt DE. Role of hyperactive cerebellum and motor cortex in Parkinson's disease. Neuroimage. 2007;35:222-33.
60. Lozza C, Marie RM, Baron JC. The metabolic substrates of bradykinesia and tremor in uncomplicated Parkinson's disease. Neuroimage. 2002;17:688-99.
61. Mure H, Hirano S, Tang CC, Isaias IU, Antonini A, Ma Y, et al. Parkinson's disease tremor-related metabolic network: characterization, progression, and treatment effects. Neuroimage. 2011;54:1244-53.
62. Playford ED, Jenkins IH, Passingham RE, Nutt J, Frackowiak RS, Brooks DJ. Impaired mesial frontal and putamen activation in Parkinson's disease: a positron emission tomography study. Annals of Neurology. 1992;32:151-61.
63. Brooks DJ, Salmon EP, Mathias CJ, Quinn N, Leenders KL, Bannister R, et al. The relationship between locomotor disability, autonomic dysfunction, and the integrity of the striatal dopaminergic system in patients with multiple system atrophy, pure autonomic failure, and Parkinson's disease, studied with PET. Brain : a journal of neurology. 1990;113 (Pt 5):1539-52.
64. Kish SJ, Shannak K, Hornykiewicz O. Uneven pattern of dopamine loss in the striatum of patients with idiopathic Parkinson's disease. Pathophysiologic and clinical implications. The New England journal of medicine. 1988;318:876-80.
65. Fearnley JM, Lees AJ. Ageing and Parkinson's disease: substantia nigra regional selectivity. Brain : a journal of neurology. 1991;114 (Pt 5):2283-301.

66. Vingerhoets FJ, Schulzer M, Calne DB, Snow BJ. Which clinical sign of Parkinson's disease best reflects the nigrostriatal lesion? Annals of Neurology. 1997;41.

67. Eickhoff SB, Nichols TE, Laird AR, Hoffstaedter F, Amunts K, Fox PT, et al. Behavior, sensitivity, and power of activation likelihood estimation characterized by massive empirical simulation. Neuroimage. 2016;137:70-85.

68. PD RS-fMRI meta and validation. NITRC database. https://www.nitrc.org/projects/pdrsfmriwutao/

69. Wang, J; Zhang, J; Zang, Y; Wu, T (2018): Supporting data for "Consistent decreased activity in the putamen in Parkinson's disease: A meta-analysis and an independent validation of resting-state fMRI" GigaScience Database. http://dx.doi.org/10.5524/100444

Figure 1

Click here to download Figure Fig 1.tif

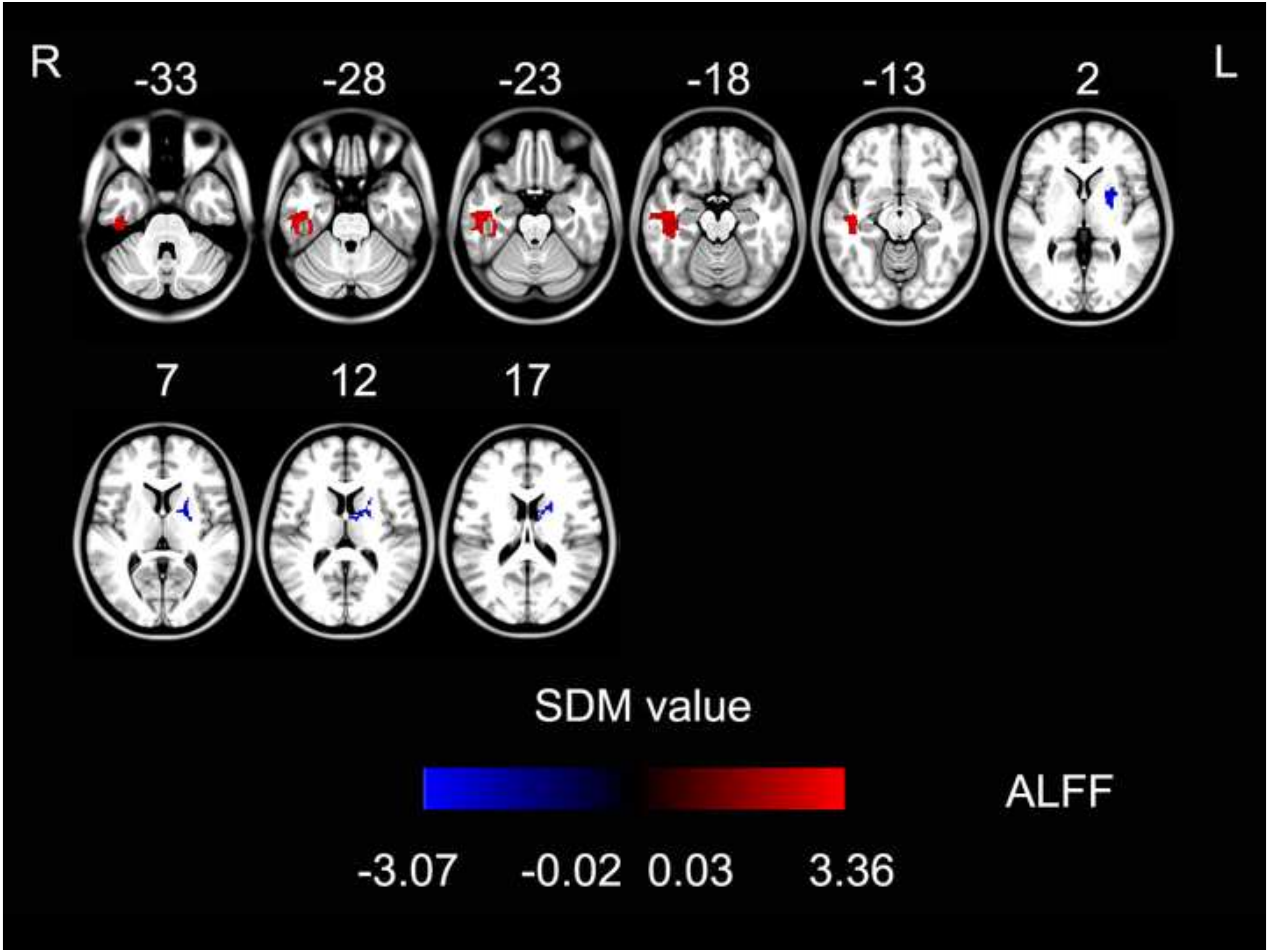

Figure 2 Click here to download Figure Fig 2.tif

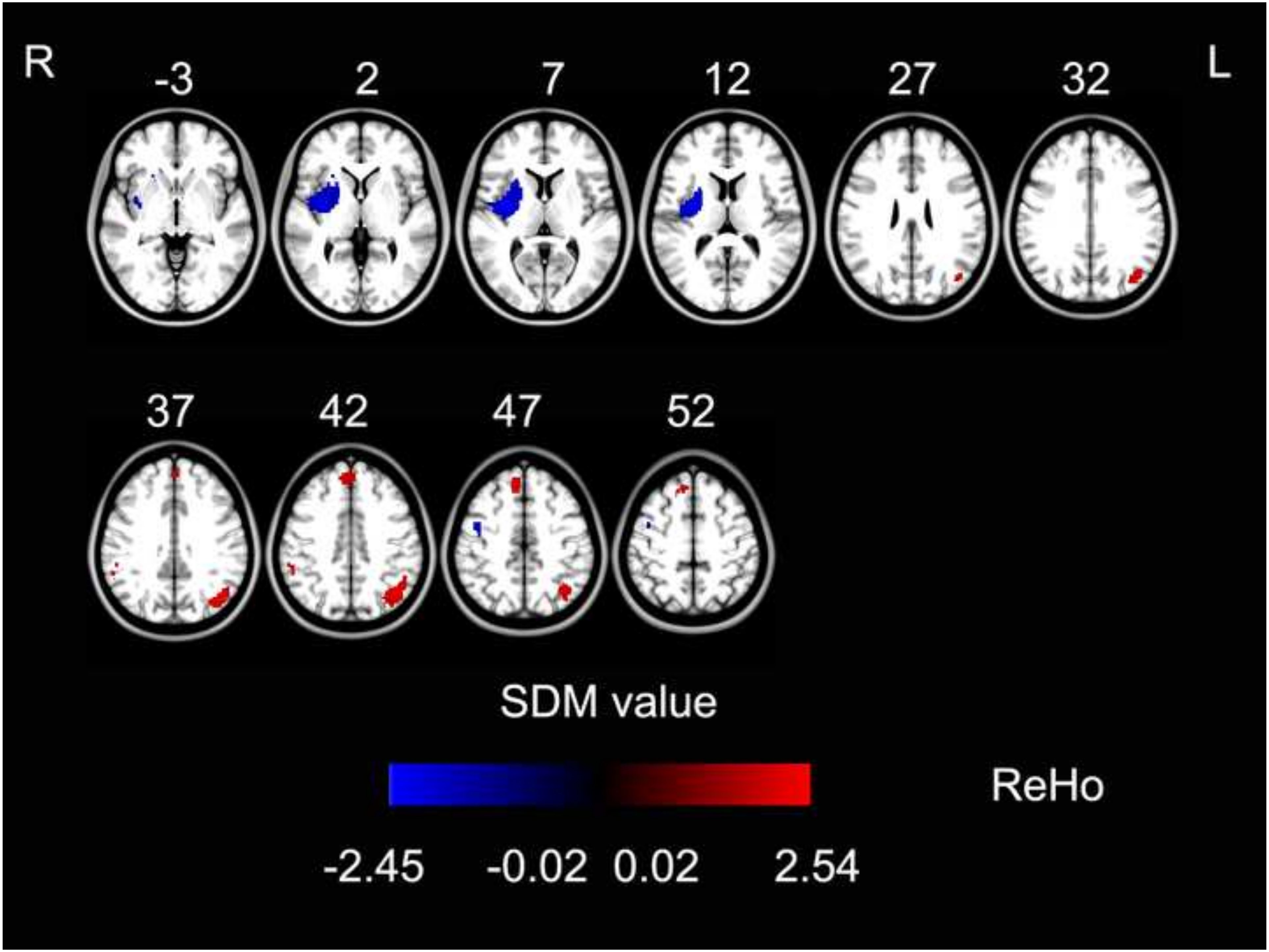

Click here to download Figure Fig 3.tif

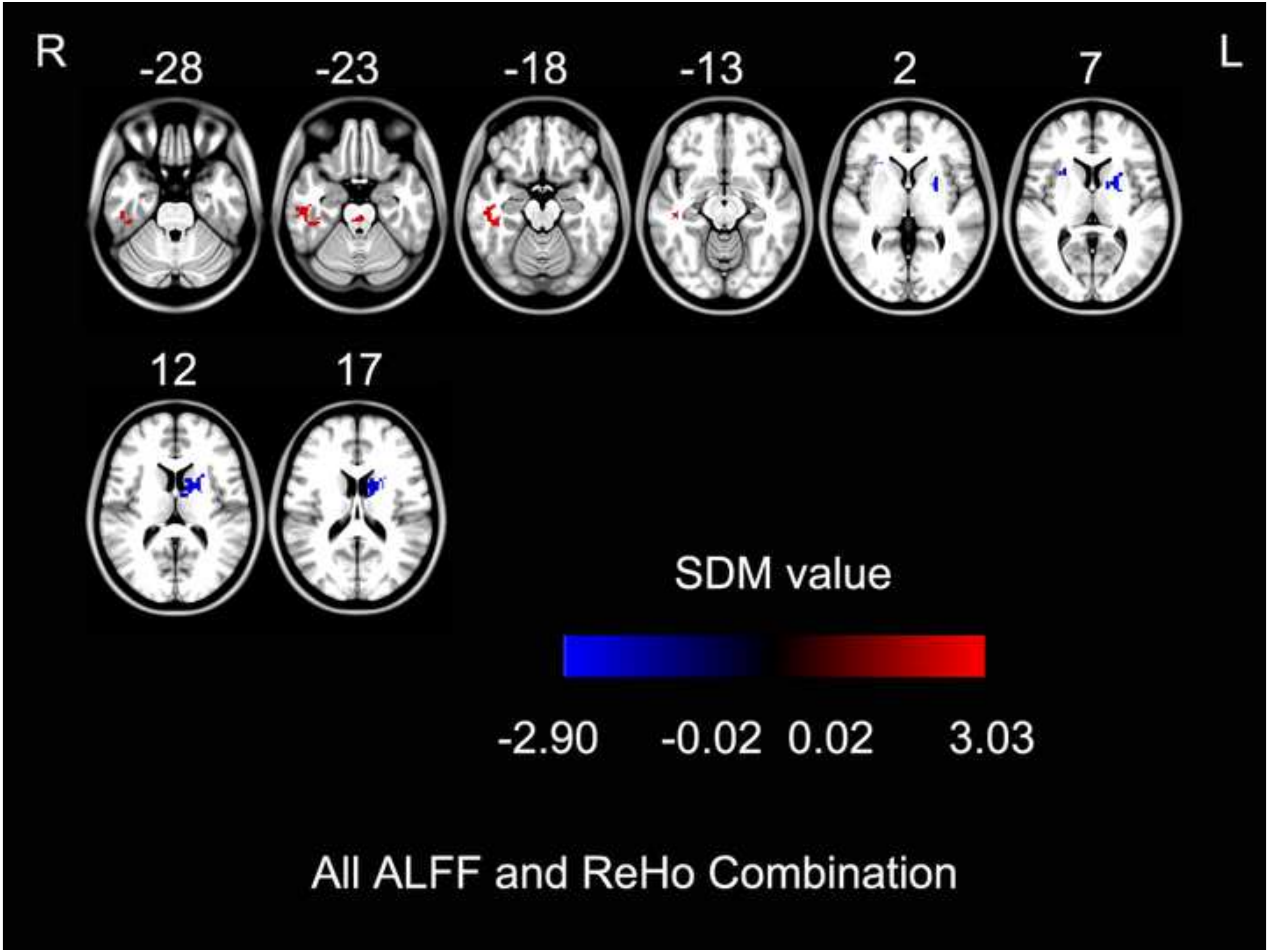

Figure 4

Click here to download Figure Fig 4.tif

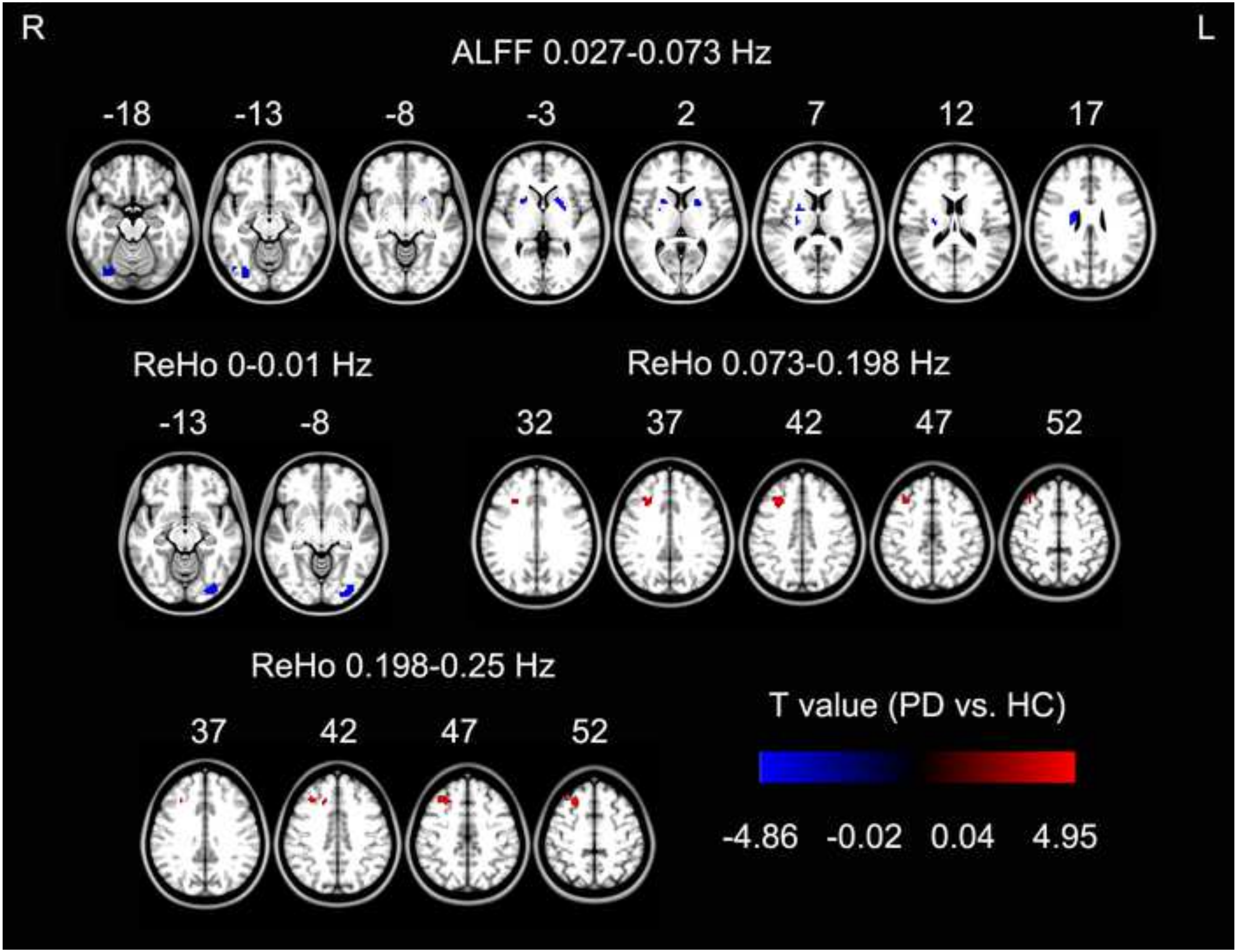

Supplement: GIGA-D-18-00065_Original_Submission.pdf [file giy071_giga-d-18-00065_original_submission.pdf]
