# Supplementary material for: Consistent decreased activity in the putamen in Parkinson's disease: a meta-analysis and an independent validation of resting-state fMRI
Source: Gigascience. 2018 Jun 18;7(6):giy071. doi: 10.1093/gigascience/giy071 (PMC6025187; doi:10.1093/gigascience/giy071)
Supplement: Reviewer_1_Report_(Original_Submission) -- Alejandro De La Vega — 03/24/2018 Reviewed [file giy071_reviewer_1_report_(original_submission).pdf]

Reviewer Report

Title: **Consistent decreased activity in the putamen in Parkinson's disease: A meta-analysis and an independent validation of resting-state fMRI**

Version: **Original Submission** **Date:** 3/24/2018

Reviewer name: **Alejandro De La Vega**

Reviewer Comments to Author:

The change of threshold for the meta-analysis suffices in my opinion to address statistical issues. In any case, I was in agreement that the previous threshold was acceptable according to the literature, so given that the results are similar with a more stringent threshold, I am OK with the inclusion of this analysis.

The authors addressed the specific points I raised in the last review. Given that the AlphaSim like algorithm in DPABI is not actually AlphaSim, I would word as follows:

Although Monte Carlo simulations in DPABI are based on AFNI's 3dClustSim, the specific algorithm used is not by the bug reported in Eklund et al., 2016 since version 1.2_141101.

Methods

Are the methods appropriate to the aims of the study, are they well described, and are necessary controls included? Yes

Conclusions

Are the conclusions adequately supported by the data shown? Yes

Reporting Standards

Does the manuscript adhere to the journal's guidelines on minimum standards of reporting? Yes

Choose an item.

Statistics

Are you able to assess all statistics in the manuscript, including the appropriateness of statistical tests used? Yes, and I have assessed the statistics in my report.

Quality of Written English

Please indicate the quality of language in the manuscript: Needs some language corrections before being published

Declaration of Competing Interests

Please complete a declaration of competing interests, considering the following questions:

- Have you in the past five years received reimbursements, fees, funding, or salary from an organisation that may in any way gain or lose financially from the publication of this manuscript, either now or in the future?
- Do you hold any stocks or shares in an organisation that may in any way gain or lose financially from the publication of this manuscript, either now or in the future?
- Do you hold or are you currently applying for any patents relating to the content of the manuscript?
- Have you received reimbursements, fees, funding, or salary from an organization that holds or has applied for patents relating to the content of the manuscript?
- Do you have any other financial competing interests?
- Do you have any non-financial competing interests in relation to this paper?

If you can answer no to all of the above, write 'I declare that I have no competing interests' below. If your reply is yes to any, please give details below.

I have no competing interests.

I agree to the open peer review policy of the journal. I understand that my name will be included on my report to the authors and, if the manuscript is accepted for publication, my named report including any attachments I upload will be posted on the website along with the authors' responses. I agree for my report to be made available under an Open Access Creative Commons CC-BY license (http://creativecommons.org/licenses/by/4.0/). I understand that any comments which I do not wish to be included in my named report can be included as confidential comments to the editors, which will not be published.

I agree to the open peer review policy of the journal

To further support our reviewers, we have joined with Publons, where you can gain additional credit to further highlight your hard work (see: https://publons.com/journal/530/gigascience). On publication of this paper, your review will be automatically added to Publons, you can then choose whether or not to claim your Publons credit. I understand this statement.

Yes Choose an item.
